# UPLC-QTOF-MS-guided isolation of anti-COPD ginsenosides from wild ginseng†

**DOI:** 10.1039/c9ra06635g

**Published:** 2019-11-26

**Authors:** Hailin Zhu, Junli Liu, Hongqiang Lin, Ying Zhang, Na Yang, Baisong Zhou, Zhongyao Wang, Alan Chen-Yu Hsu, Jinping Liu, Pingya Li

**Affiliations:** School of Pharmaceutical Sciences, Jilin University Fujin Road 1266 Changchun 130021 Jilin China liujp@jlu.edu.cn lipy@jlu.edu.cn +86-431-85619803; Research Center of Natural Drug, Jilin University Changchun 130021 Jilin China; The First Hospital of Jilin University Changchun 130021 Jilin China; Priority Research Centre for Healthy Lungs, Faculty of Health and Medicine, The University of Newcastle Newcastle NSW Australia

## Abstract

Four previously undescribed ginsenosides, along with five known analogues were isolated from wild ginseng by a UPLC-QTOF-MS-guided fractionation procedure. Their structures were elucidated on the basis of spectroscopic and spectrometric data (1D and 2D NMR, HR-ESI-MS). The isolated compounds could significantly inhibit the cigarette smoke extract (CSE)-induced inflammatory reaction in A549 cells. The HDAC2 pathway might be involved in the protective effect against the CSE-mediated inflammatory response in A549 cells.

## Abstract 2

Four previously undescribed ginsenosides, along with five known analogues were isolated from wild ginseng by a UPLC-QTOF-MS-guided fractionation procedure.

## Graphical abstract

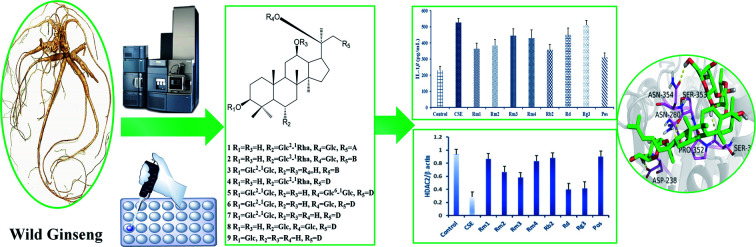

## 1 Introduction

Panax ginseng has received much attention not only as a medicinal herb but also as a functional food.^1^ Ginseng has a wide range of biological activities such as anti-inflammatory, anti-oxidant and immunomodulatory functions. It is the traditional belief that wild ginseng is more medicinally efficacious and more valuable than cultivated ginseng. In the past, natural wild ginseng has been used to refer to the original ecological ginseng. But it is hard to find the natural wild ginseng because it is nearly extinct now. Currently, according to the “Identification and Grade Quality Standards of Wild Ginseng” (National Standards of People's Republic of China, GB/T 18765-2015), wild ginseng refers to the ginseng cultivated in mountains and grown for more than 15 years in a natural environment without any human intervention. This kind of wild ginseng is a replacement for natural wild ginseng grown within the original ecological environment. It has been reported that there were differences in immunomodulating effects between wild ginseng and cultivated ginseng.^2^ In our previous study, it was also found that the wild ginseng (20 year-old mountain-cultivated ginseng) was much differentiated from cultivated ginseng. And based on UPLC-QTOF/MS, a total of 126 chemical compositions were tentatively identified or characterized from wild ginseng.^3^

Chronic Obstructive Pulmonary Disease (COPD), the fourth leading cause of death in the world currently, is usually caused by significant exposure to noxious gases or particles. The airflow limitation and persistent respiratory symptoms are the typical symptoms. Cigarette smokers have a higher prevalence of respiratory symptoms and higher COPD mortality rate, and the cigarette smoking is the leading environmental risk factor for COPD around the world.^4^ Along with the progressive lung inflammation, some pro-inflammatory mediators such as TNF-α, IL-1β and IL-6 participated in the occurrence and development of COPD.^5,6^ Ginsenosides are regarded as the main bioactive components in ginseng.^7,8^ Ginseng extract^9,10^ and monomer saponins such as Rg_1_ (ref. 11), Rh_2_ (ref. 12) and CK^13^ had been reported to significantly inhibit COPD-related inflammation *in vivo* and *in vitro*. Reduced HDAC2 activity and expression was found in COPD, resulting in amplification of the inflammatory response. Meanwhile, HDAC2 dysfunction is thought to play an important role in the development of corticosteroid resistance in COPD.^14–16^ Thus, increasing HDAC2 activity may be a promising strategy to overcome corticosteroid resistance in COPD. Existing treatments such as theophylline, nortriptyline, macrolides and selective phosphatidylinositol-3-kinase-δ inhibitors have been reported to increase HDAC2 activity effectively.^17^

As part of our continuing efforts to elucidate the chemical composition of wild ginseng furtherly and search for anti-COPD compounds from wild ginseng, a UPLC-QTOF-MS-guided fractionation procedure was performed to target ginsenosides from the anti-COPD fractions of wild ginseng, leading to the isolation of 4 previously unreported ginsenosides (1–4) and five known analogues (5–9). Herein, the isolation and structural elucidation of the isolated compounds as well as their anti-COPD activity on the CSE-stimulated A549 cells were discussed. The potential mechanism against the CSE stimulation was also preliminarily investigated in the paper.

## 2 Experimental

### 2.1 Plant materials

The 20 years-old mountain-cultivated ginsengs were collected from cultivation area in Fusong, Jilin Province, the main source of ginseng in China. The ginseng was identified by our group according to Chinese Pharmacopoeia (2015 version). The voucher specimen (no. MCG170931) was deposited at the Research Center of Natural Drug, Jilin University, Changchun, China.

### 2.2 Apparatus and chemicals

The HR-ESI-MS spectra were performed on Waters Xevo G2-XS QTOF mass spectrometer (Waters, Milford, MA, USA). NMR spectra were measured on a Bruker Avance-600 spectrometer (Bruker, Karlsruhe, Germany) in DMSO-D_6_ operating at 600 MHz for ^1^H NMR, HMBC and HMQC, 150 MHz for ^13^C NMR, respectively. Tetramethylsilane (TMS) was used as internal standard. Column chromatography was performed with silica gel (200–300 mesh) purchased from Qingdao Ocean Chemical Group Co. Ltd (Qingdao, China). Thin-layer chromatography was conducted on silica gel G plates (Qingdao Marine Chemical Inc.). Semi-preparative HPLC with 1525 binary pump combined Waters 2998 UV detector (Waters Co., Milford, MA, USA.) and YMC C_18_ column (5 μm, 20 mm × 10 mm; YMC Co., Ltd., Japan) were also used to isolate the compounds. Methanol (MeOH) and acetonitrile (ACN) were HPLC grade (Fisher, USA). Purified water was purchased from YiBao Co, Ltd (Shenzhen, China). Other solvents used were analytical grade (Beijing Chemical Works, Beijing, China).

### 2.3 Extraction and isolation

The fresh wild ginseng (1.0 kg) were crushed and extracted with 95% EtOH for three times. The extract was combined and then concentrated under vacuum to obtain the concentrated aqueous solution (0.5 L), and successively partitioned with petroleum ether, EtOAc and *n*-butanol, to afford petroleum ether (29.8 g), EtOAc (35.2 g) and *n*-butanol (133.5 g) soluble extracts, respectively. The *n*-butanol extract (inhibition of the release of pro-inflammatory mediators in CSE-induced A549 cells at 200 μM, Fig. S10A†) was subjected to rapid medium pressure purification chromatography on silica gel (200–300 mesh) eluted with a gradient of chloroform–methanol (1 : 0–0 : 1) to give 10 fractions (Fr. A–J). Fraction G showed significant inhibition of the release of pro-inflammatory mediators in CSE-induced A549 cells at 200 μM (Fig. S10B†). Subsequent UPLC-QTOF-MS-guided separation and purification targeted the subfractions of fraction G containing compounds with *m*/*z* > 500. Fraction G was further fractionated on silica gel column chromatography eluting with gradient elution of chloroform–methanol (10 : 1–1 : 1) to yield six subfractions (G1–G6, Fig. S10C†). Subfraction G5 (0.21 g) was further separated by semi-preparative RP-HPLC with ACN/H_2_O as mobile phase to obtain compound 1 (ACN–H_2_O: 19 : 81, 2 mL min^−1^, 19 min, 6 mg, 0.0006%), compound 2 (ACN–H_2_O: 19 : 81, 2 mL min^−1^, 22 min, 3 mg, 0.0003%), compound 8 (ACN–H_2_O: 21 : 79, 2 mL min^−1^, 5 min, 4 mg, 0.0003%), compound 9 (ACN–H_2_O: 42 : 58, 2 mL min^−1^, 36 min, 3 mg, 0.0003%). G4 (0.14 g) was also further separated by semi-preparative RP-HPLC with ACN/H_2_O as mobile phase to obtain compound 3 (ACN–H_2_O: 15 : 85, 2 mL min^−1^, 15 min, 4 mg, 0.0004%), compound 5 (ACN–H_2_O: 18 : 82, 2 mL min^−1^, 32 min, 3 mg, 0.0003%) and compound 6 (ACN–H_2_O: 21 : 79, 2 mL min^−1^, 13 min, 3 mg, 0.0003%). G2 (0.18 g) was also further separated by semi-preparative RP-HPLC with ACN/H_2_O as mobile phase to obtain compound 4 (ACN–H_2_O: 21 : 79, 2 mL min^−1^, 10 min, 2 mg, 0.0002%) and compound 7 (ACN–H_2_O: 28 : 72, 2 mL min^−1^, 10 min, 3 mg, 0.0003%).

### 2.4 Spectroscopic data

#### 2.4.1 Ginsenoside-Rm1 (1)

##### 6-*O*-[α-l-rhamnopyranosyl-(1→2)-β-d-glucopyranosyl]-20-*O*-β-d-glucopyranosyl-dammar-23,25-diene-3β,6α,12β,20*S*-tetraol

White amorphous powder; Libermann–Burchard reaction was positive; Molisch reaction was positive; suggesting the existence of triterpenoid structure; ^1^H and ^13^C NMR: see Table 1; HR-ESI-MS *m*/*z* 943.5270 [M − H]^−^ (calc. for C_48_H_79_O_18_ is 943.5266); HR-MS, ^1^H and ^13^C NMR, HMQC, HMBC spectra see in the ESI (Fig. S1-1–S1-5).†

**Table 1:** ^1^H and ^13^C-NMR (600 MHz, 150 MHz in DMSO-D_6_) data for 1, 2, 4

Position	1		2		4	
	*δ*_C_	*δ*_H_	*δ*_C_	*δ*_H_	*δ*_C_	*δ*_H_
1	38.73	1.44(1H, m), 0.91(1H, m)	38.88	1.41(1H, m), 0.99(1H, m)	38.76	1.32(1H, m), 0.92(1H, m)
2	26.80	1.51(1H, m), 1.50(1H, m)	26.94	1.60(1H, m), 1.47(1H, m)	26.79	1.82(1H, m), 1.75(1H, m)
3	76.90	3.54(1H, m)	77.37	3.51(1H, m)	77.21	3.65(1H, m)
4	39.98	—	39.97	—	39.91	—
5	59.86	1.40(1H, m)	60.01	1.45(1H, m)	59.84	1.07(1H, m)
6	73.84	4.66(1H, m)	72.73	4.63(1H, m)	72.63	4.53(1H, m)
7	44.75	2.17(1H, m), 1.76(1H, m)	44.90	2.14(1H, m), 1.89(1H, m)	44.75	2.24(1H, m), 1.90(1H, m)
8	40.16	—	40.31	—	40.16	—
9	48.64	1.42(1H, m)	48.79	1.39(1H, m)	48.72	1.17(1H, m)
10	38.59	—	38.73	—	38.63	—
11	30.27	1.92(1H, m), 1.41(1H, m)	30.43	2.01(1H, m), 1.41(1H, m)	30.91	2.15(1H, m), 1.07(1H, m)
12	69.08	3.86(1H, m)	70.32	3.87(1H, m)	69.81	3.90(1H, m)
13	48.06	1.69(1H, m)	48.61	1.73(1H, m)	47.03	1.96(1H, m)
14	50.80	—	50.95	—	50.94	—
15	30.09	1.40(1H, m), 0.91(1H, m)	30.24	1.37(1H, m), 0.88(1H, m)	30.73	1.37(1H, m), 0.86(1H, m)
16	25.90	1.48(1H, m), 1.26(1H, m)	26.94	1.45(1H, m), 1.26(1H, m)	26.03	1.45(1H, m), 1.07(1H, m)
17	51.05	2.50(1H, m)	51.20	2.47(1H, m)	53.49	2.30(1H, m)
18	16.74	0.82(3H, s)	16.80	0.91(3H, s)	16.97	0.83(3H, s)
19	16.84	0.81(3H, s)	16.89	0.78(3H, s)	17.56	0.83(3H, s)
20	82.02	—	82.17	—	75.24	—
21	22.37	1.22(3H, s)	22.10	1.19(3H, s)	26.38	0.97(3H, s)
22	40.01	2.13(1H, m), 1.88(1H, m)	141.31	6.05(1H, d, *J* = 11.5 Hz)	34.911	2.01(1H, m), 1.32(1H, m)
23	127.283	5.53(1H, dt, *J* = 12.5, 7.5 Hz)	127.43	6.25(1H, m)	21.973	2.60(1H, m), 2.25(1H, m)
24	134.62	6.39(1H, d, *J* = 11.0 Hz)	121.77	6.02(1H, d, *J* = 11.5 Hz)	125.517	5.48(1H, m)
25	141.72	—	134.77	—	130.16	—
26	114.63	4.98(1H, m), 4.89(1H, m)	31.12	2.25(3H, s)	25.56	1.56(3H, s)
27	17.79	1.83(3H, s)	22.52	1.91(3H, s)	16.57	1.22(3H, s)
28	30.97	1.62(3H, s)	31.12	1.92(3H, s)	31.00	1.63(3H, s)
29	17.79	0.94(3H, s)	16.99	0.99(3H, s)	16.74	0.96(3H, s)
30	16.65	0.81(3H, s)	16.80	0.78(3H, s)	16.46	0.82(3H, s)
6-Glc-1′	99.91	5.11(1H, d, *J* = 7.0 Hz)	100.06	5.09(1H, d, *J* = 7.5 Hz)	99.94	5.35(1H, m)
6-Glc-2′	77.22	4.48(1H, m)	77.37	4.40(1H, m)	77.53	4.19(1H, m)
6-Glc-3′	77.51	4.34(1H, m)	78.43	4.32(1H, m)	77.21	4.32(1H, m)
6-Glc-4′	70.82	4.14(1H, m)	70.96	4.12(1H, m)	70.85	4.12(1H, m)
6-Glc-5′	76.78	3.90(1H, brs)	77.37	3.66(1H, brs)	77.21	4.06(1H, brs)
6-Glc-6′	61.47	4.62(1H, m), 4.43(1H, m)	61.62	4.45(1H, m), 4.59(1H, m)	61.49	4.37(1H, m), 4.24(1H, m)
2′-Rham-1′′	99.91	6.32(1H, m)	100.06	6.42(1H, m)	99.87	6.59(1H, m)
2′-Rham-2′′	73.65	5.06(1H, m)	70.63	4.85(1H, m)	70.50	4.60(1H, m)
2′-Rham-3′′	70.48	4.87(1H, m)	70.32	4.84(1H, m)	70.20	4.47(1H, m)
2′-Rham-4′′	72.05	4.35(1H, m)	72.73	4.31(1H, m)	72.06	4.12(1H, m)
2′-Rham-5′′	67.84	4.88(1H, m)	67.99	4.84(1H, m)	67.88	4.94(1H, m)
2′-Rham-6′′	16.74	1.58(3H, d, *J* = 6.5 Hz)	17.94	1.60(3H, d, *J* = 6.5 Hz)	17.80	1.34(3H, d, *J* = 6.5 Hz)
20-Glc-1′′′	96.62	4.91(1H, d, *J* = 7.5 Hz)	96.77	5.03(1H, d, *J* = 7.5 Hz)	—	—
20-Glc-2′′′	73.84	3.66(1H, m)	74.00	3.59(1H, m)	—	—
20-Glc-3′′′	77.36	4.18(1H, m)	77.66	4.15(1H, m)	—	—
20-Glc-4′′′	69.28	3.84(1H, m)	69.72	3.87(1H, m)	—	—
20-Glc-5′′′	76.68	3.62(1H, m)	77.37	3.51(1H, m)	—	—
20-Glc-6′′′	61.23	4.56(1H, m), 4.26(1H, m)	61.38	4.53(1H, m), 4.23(1H, m)	—	—
–O–CO–	—	—	—	—	170.90	—
–CH_2_	—	—	—	—	38.76	3.77(2H, m)
–CO–OH	—	—	—	—	171.20	—

#### 2.4.2 Ginsenoside-Rm2 (2)

##### 6-*O*-[α-l-rhamnopyranosyl-(1→2)-β-d-glucopyranosyl]-20-*O*-β-d-glucopyranosyl-dammar-22(23),24-diene-3β,6α,12β,20*S*-tetraol

White amorphous powder; Libermann–Burchard reaction was positive; Molisch reaction was positive; suggesting the existence of triterpenoid structure; ^1^H and ^13^C NMR: see Table 1; HR-ESI-MS *m*/*z* 943.5272 [M − H]^−^ (calc. for C_48_H_79_O_18_ is 943.5266); HR-MS, ^1^H and ^13^C NMR, HMQC, HMBC spectra see in the ESI (Fig. S2-1–S2-5).†

#### 2.4.3 Ginsenoside-Rm3 (3)

##### 3-*O*-[β-d-glucopyranosyl-(1→2)-β-d-glucopyranosyl]-3β,12β,20*S*,25-tetrahydroxydammar-23-ene

White amorphous powder; Libermann–Burchard reaction was positive; Molisch reaction was positive; suggesting the existence of triterpenoid structure; ^1^H and ^13^C NMR: see Table 2; HR-ESI-MS *m*/*z* 799.4897 [M − H]^−^ (calc. for C_42_H_71_O_14_ is 799.4844); HR-MS, ^1^H and ^13^C NMR, HMQC, HMBC spectra see in the ESI (Fig. S3-1–S3-5).†

**Table 2:** ^1^H and ^13^C-NMR (600 MHz, 150 MHz in DMSO-D_6_) data for 3^a^

Position	3		Position	3	
	*δ*_C_	*δ*_H_		*δ*_C_	*δ*_H_
1	39.90	1.40(1H, m), 0.91(1H, m)	22	136.16	6.07(1H, d, *J* = 16.0 Hz)
2	25.79	2.25(1H, m), 1.55(1H, m)	23	126.20	6.31(1H, m)
3	88.22	3.15(1H, m)	24	39.50	2.76(1H, m), 2.44(1H, m)
4	39.60	—	25	80.34	—
5	55.61	0.69(1H, m)	26	29.94	1.64(3H, s)
6	17.74	1.48(1H, m), 1.21(1H, m)	27	29.96	1.56(3H, s)
7	34.42	1.36(1H, m), 1.33(1H, m)	28	27.48	0.98(3H, s)
8	40.02	—	29	15.99	0.90(3H, s)
9	49.44	1.31(1H, m)	30	16.48	0.81(3H, s)
10	36.29	—	3-Glc-1′	103.66	4.96(1H, d, *J* = 7.5 Hz)
11	30.98	1.92(1H, m), 1.63(1H, m)	3-Glc-2′	81.35	4.02(1H, m)
12	70.02	3.30(1H, m)	3-Glc-3′	76.16	3.99(1H, m)
13	48.53	1.92(1H, m)	3-Glc-4′	69.88	3.98(1H, m)
14	50.98	—	3-Glc-5′	76.43	3.16(1H, brs)
15	30.44	1.47(1H, m), 0.94(1H, m)	3-Glc-6′	61.06	4.59(1H, m), 4.42(1H, m)
16	25.79	1.58(1H, m), 1.36(1H, m)	2′-Glc-1′′	103.87	5.10(1H, d, *J* = 7.5 Hz)
17	52.97	2.30(1H, m)	2′-Glc-2′′	75.30	3.42(1H, m)
18	15.37	0.82(3H, s)	2′-Glc-3′′	76.88	4.14(1H, m)
19	15.93	0.74(3H, s)	2′-Glc-4′′	70.02	4.25(1H, m)
20	72.29	—	2′-Glc-5′′	76.55	3.42(1H, m)
21	27.48	1.02(3H, s)	2′-Glc-6′′	60.89	4.42(1H, m), 4.26(1H, m)

a Delta is ppm of chemical shifts which are reported in parts per million (*δ*), and coupling constants (*J*) are expressed in hertz.

#### 2.4.4 Ginsenoside-Rm4 (4)

##### 6-*O*-[α-l-rhamnopyranosyl-(1→2)-β-d-glucopyranosyl]-20-*O*-malonyl-dammar-24-ene-3β,6α,12β,20*S*-tetraol

White amorphous powder; Libermann–Burchard reaction was positive; Molisch reaction was positive; suggesting the existence of triterpenoid structure; ^1^H and ^13^C NMR: see Table 1; HR-ESI-MS *m*/*z* 869.4890 [M − H]^−^ (calc. for C_45_H_73_O_16_ is 869.4899); HR-MS, ^1^H and ^13^C NMR, HMQC, HMBC spectra see in the ESI (Fig. S4-1–S4-5).†

#### 2.4.5 Compound 5–9

##### Compound 5–9

White amorphous powder; Libermann–Burchard reaction was positive; Molisch reaction was positive; suggesting the existence of triterpenoid structure; ^1^H and ^13^C NMR, spectra see in the ESI (Fig. S5-1–S9-2).†

### 2.5 Bioassay

#### 2.5.1 Preparation of cigarette smoke extract

In the present study, the cigarettes used were Xiongshi cigarette (China Tobacco Zhejiang Industrial Co., Ltd, Hangzhou, China) containing 11 mg of tar, 0.7 mg of nicotine and 13 mg of carbon monoxide per cigarette. Cigarette smoke extract (CSE) was prepared essentially as reported previously.^18–20^ Smoke from one cigarette was bubbled into 20 mL of culture medium (300 s per cigarette). The CSE solution was filtered through a 0.22 μm sterile filter after being incubated at 37 °C for 30 min. The CSE solution was prepared freshly and was used within half an hour. This CSE solution was regarded as the highest concentration (100%).

#### 2.5.2 Cell viability assay

Human lung carcinoma A549 cells were obtained from the Department of Pathogen Biology, Basic Medical College, Jilin University. Each ginsenoside was dissolved in DMSO to obtain the stock solution stored in 4 °C. The final needed concentration of each compound was acquired by being diluted with Dulbecco's Modified Eagle Medium (DMEM). The growth-inhibition effect of CSE and the effect of new ginsenosides on cell viability of A549 cells were evaluated by MTT assay.^21^

A549 cells were cultured in 96-well plates at a density of 5 × 10^5^ cells per well treated with the presence of CSE (5%, 10%, 20%, 30% and 40%) for 18 h, or treated with ginsenosides at the concentrations of 0.0, 10.0, 20.0, 40.0, 80.0 μM for 24 h.

#### 2.5.3 Drug treatment

For all group, A549 cells were cultured for 18 h in 96-well plates at a density of 5 × 10^5^ cells per mL. In control group, A549 cells were cultured normally without the CSE stimulation. In CSE group, the cells were stimulated with a certain amount of CSE without any drug intervened. In ginsenoside groups, the cells were treated simultaneously with CSE and 10, 20, 40, and 80 μM of each ginsenoside.

#### 2.5.4 Enzyme-linked immunosorbent assay

IL-1β, IL-6 and TNF-α contents in the cell culture supernatant were determined with ELISA kits (Nanjing Jiancheng Bioengineering Institute) after 18 h of incubation. All procedures were performed following the manufacturer's instructions.

#### 2.5.5 Western blotting

Western blotting was performed as previous described^22^ to discuss the effects of ginsenosides on HDAC2 expression in CSE-stimulated A549 cells. Both anti-HDAC2 (#ab32117) and anti-β-actin (#ab137550) were purchased from Abcam Company (Cambridge, UK). The HDAC2 band intensities were compared with reference to β-actin control.

#### 2.5.6 Statistical analysis

Graphpad Prism 6.0 software (CA, USA) was used for all statistical analysis. The results are expressed as *x̄* ±SD. Statistical significance was calculated with two tailed test or a one-way analysis of variance (ANOVA), and *p*-value < 0.05 was considered as statistically significant.

#### 2.5.7 Molecular docking assay

The GLIDE 6.7 software and Maestro Elements 2.2 software (Schrödinger, New York, NY, USA) were applied to perform molecular docking. Firstly, the 3D structures of ginsenosides were acquired respectively; secondly, the ginsenosides' bond angles and orders were then assigned using LigPrep module; thirdly, the 3D X-ray crystal structure of HDAC2 (PDB ID: 5IWG) was retrieved from Protein Data Bank (PDB) and was optimized according to the processes previous reported.^23^ Fourthly, the docking were performed with the following main parameters: the energy of conjugate of ligand and receptor was minimized to a root-mean-square deviation of 0.30 Å in optimized potentials for liquid simulations 3 force field, and the grid box at active site was set at the size of 20 Å. Finally, the highest XPG score pose was chosen as the dominant binding form and the visual analysis was performed by PyMol (Schrödinger) software.

## 3 Results and discussion

### 3.1 Structure elucidation

The 95% EtOH extract of wild ginseng was suspended in aqueous solution and partitioned with petroleum ether, EtOAc and *n*-butanol. UPLC-QTOF-MS-guided isolation of bioactive *n*-butanol soluble extracts (inhibition of the release of pro-inflammatory mediators in CSE-induced A549 cells at 200 μM) led to identify nine ginsenosides (Fig. S10A†). Five known compounds were identified as ginsenoside Rb2 (5),^24^ Rd (6),^24^ Rg3 (7),^25^ Rg1 (8)^25^ and Rh2 (9)^26^ by comparing spectroscopic data with those of reported values.

**Fig. 1:**
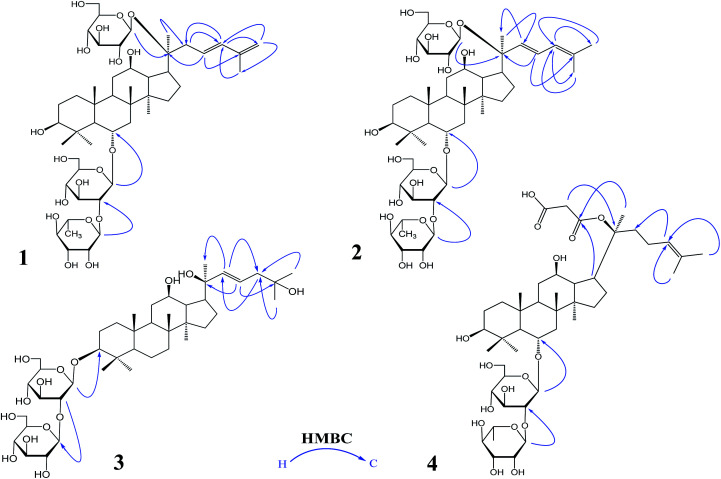
The important HMBC correlations of 1–4: ginsenoside Rm1 (1); ginsenoside Rm2 (2); ginsenoside Rm3 (3); ginsenoside Rm4 (4) HMBC: heteronuclear multiple bond correlation.

**Fig. 2:**
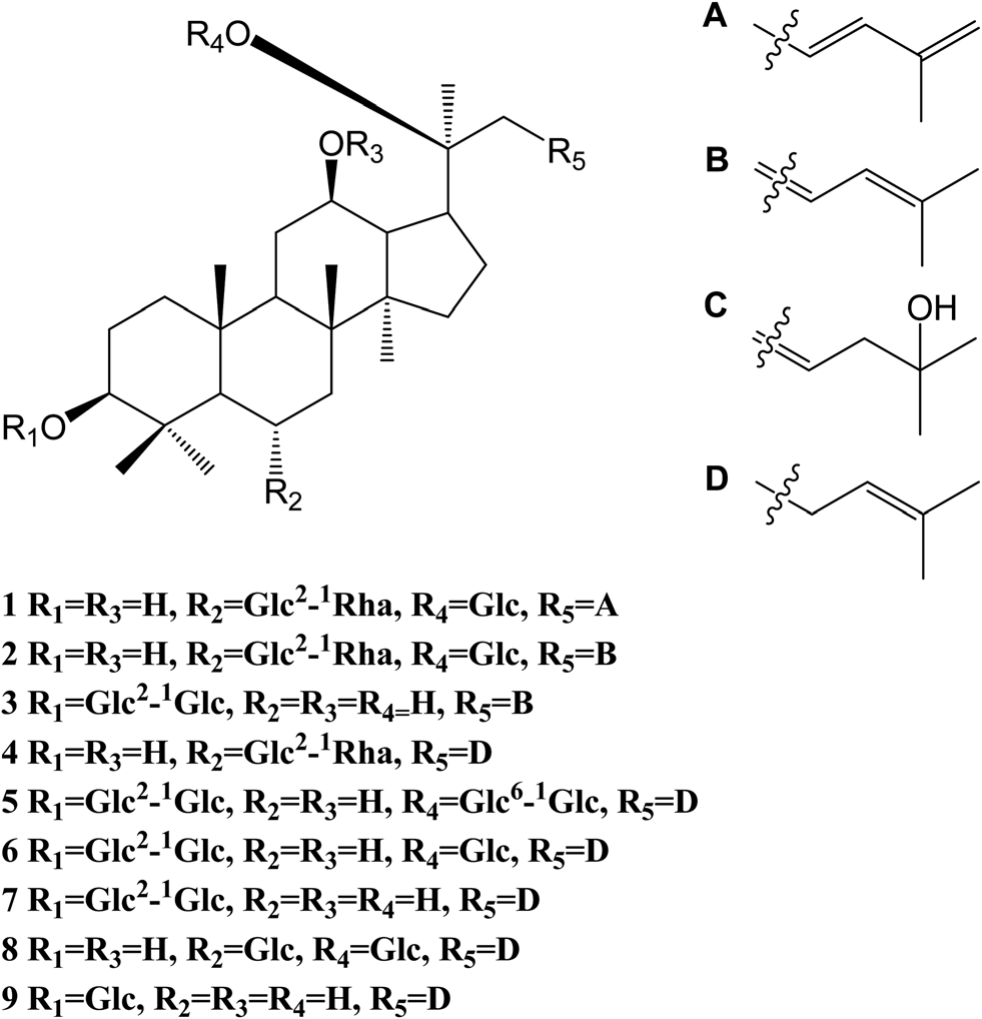
Chemical structures of 1–9: ginsenoside Rm1 (1); ginsenoside Rm2 (2); ginsenoside Rm3 (3); ginsenoside Rm4 (4); ginsenoside Rb2 (5); ginsenoside Rd (6); ginsenoside Rg3 (7); ginsenoside Rg1 (8); ginsenoside Rh2 (9).

Compound 1 was a white amorphous powder (MeOH). The molecular formula was established as C_48_H_80_O_18_ by combining NMR spectra and HR-ESI-MS at *m*/*z* 943.5270 [M − H]^−^ (calc. for 943.5266). The ^1^H NMR spectrum of 1 displayed eight methyl signals at *δ* 0.81 (3H, s), 0.81 (3H, s), 0.82 (3H, s), 0.94 (3H, s), 1.22 (3H, s), 1.58 (3H, d, *J* = 6.5 Hz), 1.62 (3H, s) and 1.83 (3H, s). The ^13^C NMR spectrum of 1 showed 48 signals, including ten methylene [two of them bearing an oxygen atom respectively (*δ* 61.23, 61.47), and one of them was olefinic signal at *δ* 114.63], twenty-four methine [eighteen of them bearing an oxygen atom respectively (*δ* 67.84, 69.08, 69.28, 70.48, 70.82, 72.05, 73.65, 73.84, 73.84, 76.68, 76.78, 76.90, 77.22, 77.36, 77.51, 96.62, 99.91 and 99.91), and two of them were olefinic signals at *δ* 127.28 and 134.62], six quaternary carbon [*δ* 38.59, 39.98, 40.16, 50.80, 82.02 and an olefinic signal at *δ* 141.72], eight methyl carbons (*δ* 16.65, 16.74, 16.74, 16.84, 17.79, 17.79, 22.37 and 30.97). There is a resemblance with the chemical shifts of 1 along with ginsenoside Re^27^ except the signals of the side chain. The main differences were the replacement of two olefinic carbons by four olefinic carbons (*δ* 141.72, 134.62, 127.28 and 114.63), and only two methyl signals (*δ* 22.37 and 17.79) in 1. Furthermore, comparison of the NMR spectroscopic data of 1 with those of cylindrictone A,^28^ whose structure was 12β,20β-dihydroxy-23,25-diene-3-oxo-dammarane, a 23,25-diene group was deduced to exist in compound 1. The location of this group was confirmed by the heteronuclear multiple bond coherence (HMBC) spectrum of 1. The correlations between H-22 with C-21 and C-24, H-23 with C-20 and C-25, H-24 with C-22, C-26 and C-27, H-26 with C-24 and C-27, H-27 with C-24 and C-26, were observed in the HMBC spectrum of 1. The structure of 1 were deduced as 6-*O*-[α-l-rhamnopyranosyl-(1→2)-β-d-glucopyranosyl]-20-*O*-β-d-glucopyranosyl-dammar-23,25-diene-3β,6α,12β,20*S*-tetraol (ginsenoside Rm1, Fig. 1). Compound 1 was a minor glucoside in wild ginseng. The carbon and proton signals of 1 were fully assigned on the basis of HMQC and HMBC spectra (Table 1 and Fig. 2).

Compound 2 was a white amorphous powder (MeOH). The molecular formula was established as C_48_H_80_O_18_ by combining the NMR spectra and HR-ESI-MS at *m*/*z* 943.5272 [M − H]^−^ (calc. for 943.5266). The ^1^H NMR spectrum of 2 displayed nine methyl signals at *δ* 0.78 (3H, s), 0.78 (3H, s), 0.91 (3H, s), 0.99 (3H, s), 1.19 (3H, s), 1.60 (3H, d, *J* = 6.5 Hz), 1.91 (3H, s), 1.92 (3H, s) and 2.25 (3H, s). The ^13^C NMR spectrum of 2 revealed 48 signals, including eight methylene [two of them bearing an oxygen atom respectively (*δ* 61.38, 61.62)], twenty-five methine [eighteen of them bearing an oxygen atom respectively (*δ* 67.99, 69.72, 70.32, 70.32, 70.63, 70.96, 72.73, 72.73, 74.00, 77.37, 77.37, 77.37, 77.37, 77.66, 78.43, 96.77, 100.06 and 100.06), and three of them were olefinic signals at *δ* 121.77, 127.43 and 141.31], six quaternary carbon [*δ* 39.97, 40.31, 38.73, 50.95, 82.17 and an olefinic signal at *δ* 134.77], nine methyl carbons (*δ* 16.80, 16.80, 16.89, 16.99, 17.94, 22.10, 22.52, 31.12 and 31.12). There is a resemblance with the chemical shifts of 2 along with ginsenoside Re^27^ or compound 1 except the signals of the side chain. The main differences were the four olefinic carbons (*δ* 121.77, 127.43, 134.77 and 141.31), and three methyl signals (*δ* 22.10, 22.52 and 31.12) in 2. Furthermore, comparison of the NMR spectroscopic data of 2 with those of notoginsenoside ST14,^29^ whose structure was (3β,6α,12β,20*S*,22*E*)-3,6,12,20-tetrahydroxydammar-22(23),24-diene-6-*O*-β-d-glucopyranoside, a 22(23),24-diene group was deduced to exist in compound 2. The location of this group was confirmed by HMBC of 2. In HMBC spectrum, the correlations between H-22 with C-21 and C-24, H-23 with C-20 and C-25, H-24 with C-22, C-26 and C-27, H-26 with C-24 and C-27, H-27 with C-24 and C-26, were observed. Thus, the structure of compound 2 was identified as 6-*O*-[α-l-rhamnopyranosyl-(1→2)-β-d-glucopyranosyl]-20-*O*-β-d-glucopyranosyl-dammar-22(23), 24-diene-3β,6α,12β,20*S*-tetraol (ginsenoside Rm2, Fig. 1). Compound 2 was also a minor glucoside in wild ginseng. The complete proton and carbon signal assignments were shown in Table 1.

Compound 3, a white amorphous powder (MeOH), possessed a molecular formula of C_42_H_72_O_14_, as elucidated by combining the NMR spectra and HR-ESI-MS at *m*/*z* 799.4897 [M − H]^−^ (calc. for 799.4844). The ^1^H NMR spectrum of 3 displayed eight methyl signals at *δ* 0.74 (3H, s), 0.81 (3H, s), 0.82 (3H, s), 0.90 (3H, s), 0.98 (3H, s), 1.02 (3H, s), 1.56 (3H, s) and 1.64 (3H, s). The ^13^C NMR spectrum of 3 revealed 42 signals, including ten methylene [two of them bearing an oxygen atom respectively (*δ* 60.89, 61.06)], twenty methine [twelve of them bearing an oxygen atom respectively (*δ* 69.88, 70.02, 70.02, 75.30, 76.55, 76.16, 76.43, 76.88, 81.35, 88.22, 103.6 and 103.87), and two of them were olefinic signals at *δ* 126.20 and 136.16], six quaternary carbon [*δ* 36.29, 39.60, 40.02, 50.98, 72.29 and 80.34], eight methyl carbons (*δ* 15.37, 15.93, 15.99, 16.48, 27.48, 27.48, 29.94 and 29.96). The NMR data of 3 were similar to those of quinquenoside A,^29,30^ whose structure was 3-*O*-[β-d-glucopyranosyl-(1→2)-β-d-glucopyranosyl]-20-*O*-[β-d-glu-copyranosyl-(1→6)-β-d-glucopyranosyl]-3β,12β,20*S*,25-tetrahydroxydammar-23-ene, except the absence of β-d-glucopyranosyl-(1→6)-β-d-glucopyranosyl group signals in 3. And the chemical shift of C-20 was changed to *δ* 72.29, indicated that C-20 was linked to a free hydroxyl group instead of being glycosylated. The location of hydroxyl group was determined by HMBC of 3. In HMBC spectrum, the correlations between H-22 with C-21 and C-24, H-23 with C-20 and C-25, were observed. Thus, the structure of compound 3 was identified as 3-*O*-[β-d-glucopyranosyl-(1→2)-β-d-glucopyranosyl]-3β,12β,20*S*,25-tetrahydroxydammar-23-ene (ginsenoside Rm3, Fig. 1). Compound 3 was also a minor glucoside in wild ginseng. The complete signal assignments on basis of HMQC and HMBC were shown in Table 1.

Compound 4, a white amorphous powder (MeOH), possessed a molecular formula of C_45_H_74_O_16_, as elucidated by the NMR spectra and HR-ESI-MS at *m*/*z* 869.4890 [M − H]^−^ (calc. for 869.4899). The ^1^H NMR spectrum of 4 displayed eight methyl signals at *δ* 0.82 (3H, s),0.83 (3H, s), 0.83 (3H, s), 0.96 (3H, s), 0.97 (3H, s), 1.22 (3H, s), 1.34 (3H, d, *J* = 6.5 Hz), 1.56 (3H, s) and 1.63(3H, d, *J* = 4.5 Hz). The ^13^C NMR spectrum of 4 revealed 45 signals, including ten methylene [one of them bearing an oxygen atom (*δ* 61.49)], eighteen methine, eight quaternary carbons [*δ* 38.63, 39.91, 40.16, 50.94, 75.24, 130.16, 170.90 and 171.20], eight methyl carbons (*δ* 16.46, 16.57, 16.74, 17.56, 17.80, 25.56, 26.38 and 31.00). The NMR data of 4 were similar to those of ginsenoside Rg_2_,^31^ except a malonyl group was appeared in 4. The location of the malonyl group was determined by HMBC of 4. The following correlations were found: H-21 with C-17 and C=O, H-17 with C-21 and C=O, which indicated that the malonyl group was linked to C-20. Thus, the structure of 4 was identified as 6-*O*-[α-l-rhamnopyranosyl-(1→2)-β-d-glucopyranosyl]-20-*O*-malonyl-dammar-24-ene-3β,6α,12β,20*S*-tetraol (ginsenoside Rm4, Fig. 1). Compound 4 was also a minor glucoside in wild ginseng. The proton and carbon signals of 4 were fully assigned on the basis of HMQC and HMBC spectra (Table 1).

### 3.2 Bioactivity evaluation

Ginsenoside Rg_1_ (ref. 11) and Rh_2_ (ref. 12) had been reported to significantly inhibit COPD-related inflammation. As part of an ongoing effort to search for anti-COPD agents from wild ginseng,^23^ compounds 1–7 were evaluated for their anti-inflammatory effects on CSE-induced A549 cells. Dexamethasone was selected as positive drug (Pos).^32^

#### 3.2.1 Cytotoxicity of cigarette smoke extract (CSE) and compounds 1–7 on the viability of A549 cells

**Fig. 3:**
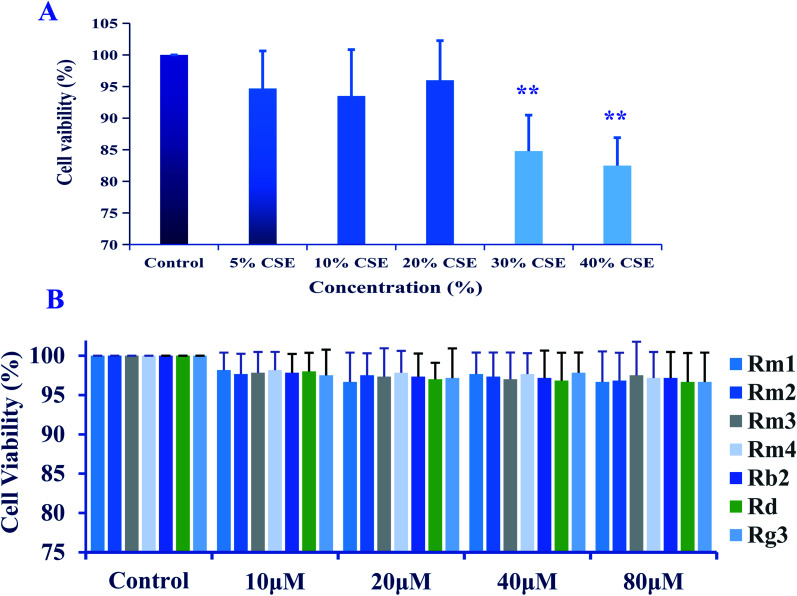
(A) Cytotoxicity of cigarette smoke extract (CSE) on A549 cells; (B) effect of compound 1–7 on the cell viability of A549 cells. The results represented as mean ± S.D. (*n* = 6). **p < 0.01, compared with control group.

The results of MTT showed that the cell viability of A549 cells was significantly affected (*p* < 0.01) by CSE (≧30%) (Fig. 3A). Therefore, in subsequent experiments, 20% CSE was chosen as stimulation. As shown in Fig. 3B, the cell viabilities of A549 cells were not significantly affected by 1–7 at 10 μM–80 μM. Then we evaluated the anti-inflammatory effects of 1–7 at 10 μM–80 μM on CSE-stimulated A549 cells.

#### 3.2.2 Effect of compounds 1–7 on CSE-induced pro-inflammatory cytokine levels in A549 cells

**Fig. 4:**
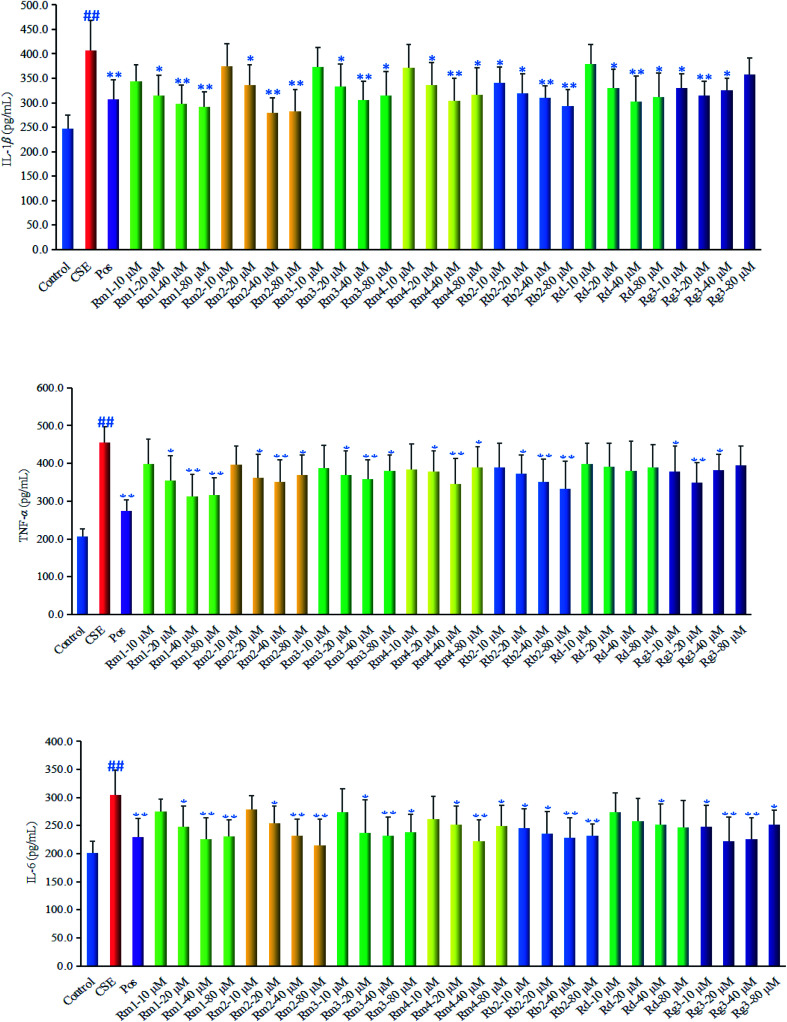
Anti-inflammatory effect of compound 1–7 (10 μM, 20 μM, 40 μM and 80 μM) on the inflammatory cytokine tumor necrosis factor-α (TNF-α), interleukin-1β (IL-1β) and interleukin-6 (IL-6) in CSE-exposed A549 cells. The results are expressed as mean ± S.D., *n* = 6. ^##^*p* < 0.01, compared with control group; **p < 0.01, compared with CSE group; *p < 0.05, compared with CSE group.

The inflammatory development is characterized by the release of pro-inflammatory mediators such as tumor necrosis factor-α (TNF-α), interleukin-1β (IL-1β) or interleukin-6 (IL-6), *etc.* Whether compounds 1–7 could inhibit the release of chemokine TNF-α, IL-1β and IL-6 from CSE-induced lung epithelial cells was investigated in this paper. As shown in Fig. 4, TNF-α, IL-1β and IL-6 production markedly increase in the cell treated with CSE (*p* < 0.01), however, treated with compounds 1–7 could significantly reduce the levels of pro-inflammatory factors to various extents. Compound 1 (Rm1) could significantly reduce TNF-α, IL-1β and IL-6 at 20 (*p* < 0.05), 40 and 80 (*p* < 0.01) μM. Compound 2 (Rm2) could significantly reduce IL-1β and IL-6 at 20 (*p* < 0.05), 40 and 80 (*p* < 0.01) μM, significantly reduce TNF-α at 20 and 80 (*p* < 0.05), 40 (*p* < 0.01) μM. Compound 3 (Rm3) and Compound 4 (Rm4) could significantly reduce IL-1β, IL-6 and TNF-*α* at 20 and 80 (*p* < 0.05), 40 (*p* < 0.01) μM. Compound 5 (Rb2) could significantly reduce IL-1β and IL-6 at 10 and 20 (*p* < 0.05), 40 and 80 (*p* < 0.01) μM, reduce TNF-α at 20 (*p* < 0.05), 40 and 80 (*p* < 0.01) μM. Compound 6 (Rd) could significantly reduce IL-1β at 20 and 80 (*p* < 0.05), 40 (*p* < 0.01) μM, reduce IL-6 at 80 (*p* < 0.05) μM, while had no significant effect on TNF-α level. Compound 7 (Rg3) could significantly reduce IL-1β and TNF-*α* at 10 and 40 (*p* < 0.05), 20 (*p* < 0.01) μM, reduce IL-6 at 10 and 80 (*p* < 0.05), 20 and 40 (*p* < 0.01) μM. The lowest effective concentration of compound 1, 2, 3, 4, 6 was all 20 μM, the lowest effective concentration of compound 5, 7 was both 10 μM. Meanwhile, the dose-dependent effect of the compounds was appeared with a certain range of 10–80 μM. Interestingly, the activity of these compounds was not always increased with the increasing concentration, namely, when the concentration arrived a certain level, the activity might decrease to some extent.

#### 3.2.3 Effect of compounds 1–7 on CSE-Mediated protein expression of HDAC2 in *vitro*

It has been demonstrated that histone deacetylase 2 (HDAC2) activity and egg self-expression in alveolar macrophages, peripheral lung tissues and bronchial tissues of COPD patients are significantly decreased.^33,34^ CSE can reduce HDAC2 activity and protein expression in rats,^35^ which can activate rat lung tissue NF-*k*B pathway.^36^ Other studies have shown that CSE exposure can cause the decrease of HDAC2 protein and activity in A549 cell nucleus^37^ and activate the NF-*k*B pathway *in vivo*.^36^

**Fig. 5:**
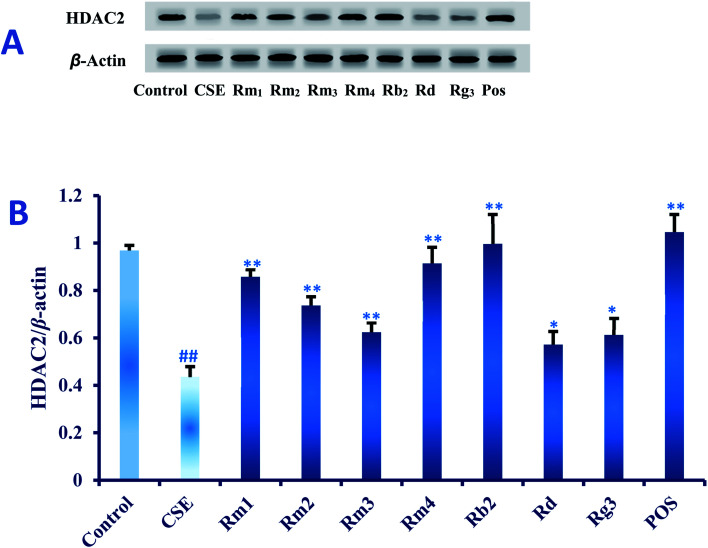
(A) Effect of compound 1–7 on the protein expression of histone deacetylase 2 (HDAC2) in the CS-induced A549 cells examined by western blot analysis (B) quantitative analyses of HDAC2 in each group. Data are expressed as mean ± SD (*n* = 6).^##^*p* < 0.01 as compared with control group, *p < 0.05, **p < 0.01 as compared with CSE group.

In order to explore the underlying mechanism of anti-inflammatory property of compounds 1–7, we investigated the effect of them on the protein expression of HDAC2 in CSE-exposed A549 cells. In the present study, after 18 hours of CSE exposure in A549 cells, the expression of HDAC2 protein in each group of 40 μM concentration was measured as shown in Fig. 5. The results showed that the level of HDAC2 protein in A549 cells decreased significantly (*p* < 0.01) after CSE 18 hours exposure. Interestingly, treatment with each ginsenoside could significantly activated the expression of HDAC2 (*p* < 0.01, *p* < 0.05) as compared to the CSE group (Fig. 5) in detail, which suggested that these compounds may up-regulated the expression of HDAC2 in CSE-exposed A549 cells.

#### 3.2.4 Molecular docking study

**Fig. 6:**
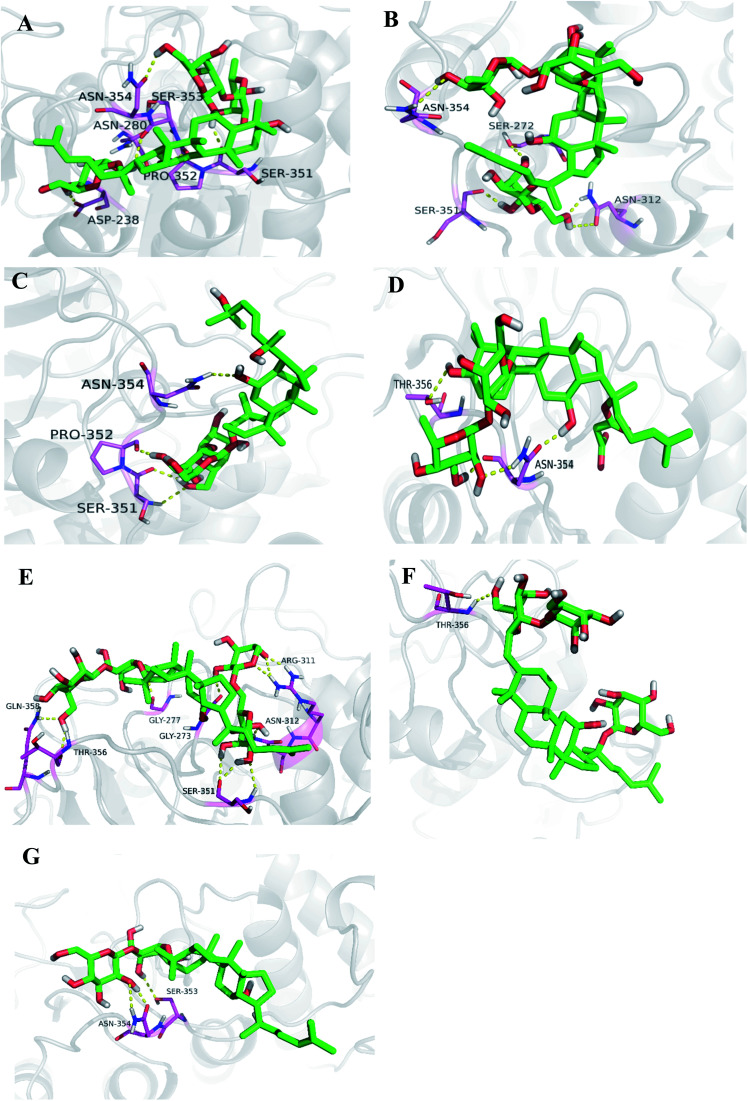
Target of compound 1–7 against CSE-stimulated inflammatory response in A549 cells. (A–G) Interaction mode of compound 1–7 within binding pocket of HDAC2. (A–G) The hydrogen bonds represented as the yellow dashed lines.

The interactions between each ginsenoside and HDAC2 were visualized in molecular docking study. Ginsenoside Rm1 towards HDAC2 (PDB ID: 5IWG) gave six hydrogen bonds between Rm1 and six residues (ASN-354, ASN-280, SER-353, SER-351, PRO-352 and ASP-238) (Fig. 6A), Rm2 towards HDAC2 (PDB ID: 5IWG) displayed five hydrogen bonds between Rm2 and four residues (ASN-354, SER-351, ASN-312 and SER-272) (Fig. 6B), Rm3 towards HDAC2 (PDB ID: 5IWG) showed four hydrogen bonds between Rm3 and three residues (ASN-354, SER-351 and PRO-352) (Fig. 6C), Rm4 towards HDAC2 (PDB ID: 5IWG) exhibited four hydrogen bonds between Rm4 and two residues (THR-356 and ASN-354) (Fig. 6D). Rb_2_ towards HDAC2 (PDB ID: 5IWG) exhibited seven hydrogen bonds between Rb_2_ and seven residues (ARG-311, ASN-312, SER-351, GLY-273, GLY-277, THR-356, GLN-358) (Fig. 6E), Rd towards HDAC2 (PDB ID: 5IWG) exhibited one hydrogen bonds between Rd and one residues (THR-356) (Fig. 6F), Rg_3_ towards HDAC2 (PDB ID: 5IWG) exhibited two hydrogen bonds between Rg_3_ and two residues (SER-353 and ASN-354) (Fig. 6G). These results were consistent with the anti-inflammatory effects of the seven ginsenosides in CSE-induced inflammatory response in A549 cells. These docking results illustrated that the ginsenosides may directly bind to HDAC2 to exert lung protective effects due to strong hydrogen bonding effects.

## 4 Conclusions

In the present study, four minor previously undescribed ginsenosides, named as ginsenoside Rm1 (1), Rm2 (2), Rm3 (3) and Rm4 (4), along with five known ginsenosides, Rb_2_ (5), Rd (6), Rg_3_ (7), Rg_1_ (8) and Rh_2_ (9) were isolated from wild ginseng by a UPLC-QTOF-MS-guided fractionation procedure. Their structures were elucidated on the basis of spectroscopic and spectrometric data (1D and 2D NMR, IR, and HR-ESI-MS). The anti-COPD activities were investigated using CSE-stimulated A549 cells. Compounds 1–7 could ameliorate inflammatory reaction *in vitr*o. Furthermore, the protective effect against CSE could be related to the HDAC2 pathway. The study provided some evidences to further elucidate the chemical composition of wild ginseng, and revealed that the compounds isolated from wild ginseng had the protective effect on the injury of cigarette smoke *in vitro*. The study provides a theoretical basis for the further utilization and development of the wild ginseng. It reminds us that it is meaningful to explore the protective effect of the wild ginseng against the COPD *in vivo*.

## Conflicts of interest

The authors declare no conflicts of interest.

## Supplementary Material

RA-009-C9RA06635G-s001
