# Supplementary material for: UPLC-QTOF-MS-guided isolation of anti-COPD ginsenosides from wild ginseng
Source: RSC Adv. 2019 Nov 26;9(66):38658–68. doi: 10.1039/c9ra06635g (PMC9075943; doi:10.1039/c9ra06635g)
Supplement: RA-009-C9RA06635G-s001 [file RA-009-C9RA06635G-s001.pdf]

Electronic Supplementary Material (ESI) for RSC Advances.
This journal is © The Royal Society of Chemistry 2019

SUPPLEMENTARY MATERIAL

UPLC-QTOF-MS-guided isolation of anti-COPD ginsenosides from Wild Ginseng

Hailin Zhu[a], Junli Liu[a], Hongqiang Lin[a], Ying Zhang[a,c], Na Yang[a], Baisong Zhou[a], Zhongyao Wang[a], Alan Chen-Yu Hsu[d], Jinping Liu[a,b*], Pingya Li[a*]

[a]School of Pharmaceutical Sciences, Jilin University, Changchun 130021, Jilin, China

[b]Research Center of Natural Drug, Jilin University, Changchun 130021, Jilin, China

[c]The First Hospital of Jilin University, Changchun 130021, Jilin, China

[d]Priority Research Centre for Healthy Lungs, Faculty of Health and Medicine, The University of Newcastle, Newcastle, NSW, Australia

[*]Co-Correspondence authors: Prof. Jinping Liu and Prof. Pingya Li, School of Pharmaceutical Sciences, Jilin University, Fujin Road 1266, Changchun 130021, China, E-mail: liujp@jlu.edu.cn (J.L.); lipy@jlu.edu.cn (P.L.) Tel.: +86-431-85619803 (J.L.)

Contents of Supplementary Information

Figure S1-1. ^{1}H NMR spectrum of Rm1 in DMSO-D_6 (600 MHz).
Figure S1-2. ^{13}C-DEPT NMR spectrum of Rm1 in DMSO-D_6 (150 MHz).
Figure S1-3. Heteronuclear multiple quantum correlation (HMQC) spectrum of Rm1.
Figure S1-4. Heteronuclear multiple bond coherence (HMBC) spectrum of Rm1.
Figure S1-5. High resolution electrospray ionization mass spectroscopy (HRESIMS) spectrum of MS^E fragment ions of Rm1.
Figure S2-1. ^{1}H NMR spectrum of Rm2 in DMSO-D_6 (600 MHz).
Figure S2-2. ^{13}C-DEPT NMR spectrum of Rm2 in DMSO-D_6 (150 MHz).
Figure S2-3. Heteronuclear multiple quantum correlation (HMQC) spectrum of Rm2.
Figure S2-4. Heteronuclear multiple bond coherence (HMBC) spectrum of Rm2.
Figure S2-5. High resolution electrospray ionization mass spectroscopy (HRESIMS) spectrum of MS^E fragment ions of Rm2.
Figure S3-1. ^{1}H NMR spectrum of Rm3 in DMSO-D_6 (600 MHz).
Figure S3-2. ^{13}C-DEPT NMR spectrum of Rm3 in DMSO-D_6 (150 MHz).
Figure S3-3. Heteronuclear multiple quantum correlation (HMQC) spectrum of Rm3.
Figure S3-4. Heteronuclear multiple bond coherence (HMBC) spectrum of Rm3.
Figure S3-5. High resolution electrospray ionization mass spectroscopy (HRESIMS) spectrum of MS^E fragment ions of Rm3.
Figure S4-1. ^{1}H NMR spectrum of Rm4 in DMSO-D_6 (600 MHz).
Figure S4-2. ^{13}C-DEPT NMR spectrum of Rm4 in DMSO-D_6 (150 MHz).
Figure S4-3. Heteronuclear multiple quantum correlation (HMQC) spectrum of Rm4.
Figure S4-4. Heteronuclear multiple bond coherence (HMBC) spectrum of Rm4.

Figure S4-5. High resolution electrospray ionization mass spectroscopy (HRESIMS) spectrum of MS^E fragment ions of Rm4.
Figure S5-1. ^1H NMR spectrum of Rb2 in Pyridine-D_5(600 MHz).
Figure S5-2. ^{13}C-DEPT NMR spectrum of Rb2 in Pyridine-D_5 (150 MHz).
Figure S6-1. ^1H NMR spectrum of Rd in Pyridine-D_5(600 MHz).
Figure S6-2. ^{13}C-DEPT NMR spectrum of Rd in Pyridine-D_5 (150 MHz).
Figure S7-1. ^1H NMR spectrum of Rg3 in DMSO-D_6 (600 MHz).
Figure S7-2. ^{13}C-DEPT NMR spectrum of Rg3 in DMSO-D_6 (150 MHz).
Figure S8-1. ^1H NMR spectrum of Rg1 in DMSO-D_6 (600 MHz).
Figure S8-2. ^{13}C-DEPT NMR spectrum of Rg1 in Pyridine-D_6 (150 MHz).
Figure S9-1. ^1H NMR spectrum of Rh2 in DMSO-D_6 (600 MHz).
Figure S9-2. ^{13}C-DEPT NMR spectrum of Rh2 in DMSO-D_6 (150 MHz).
Figure S10. Anti-inflammatory effect of petroleum ether soluble extracts (PEe), ethyl acetate soluble extracts(ASe) , n-butanol soluble extracts(BSe) (200 μM)(A), FrA-J(B) and G1-6(C) on the inflammatory cytokine tumor necrosis factor-α (TNF-*α*), interleukin-1*β* (IL-1*β*) and interleukin-6 (IL-6) in CSE-exposed A549 cells.

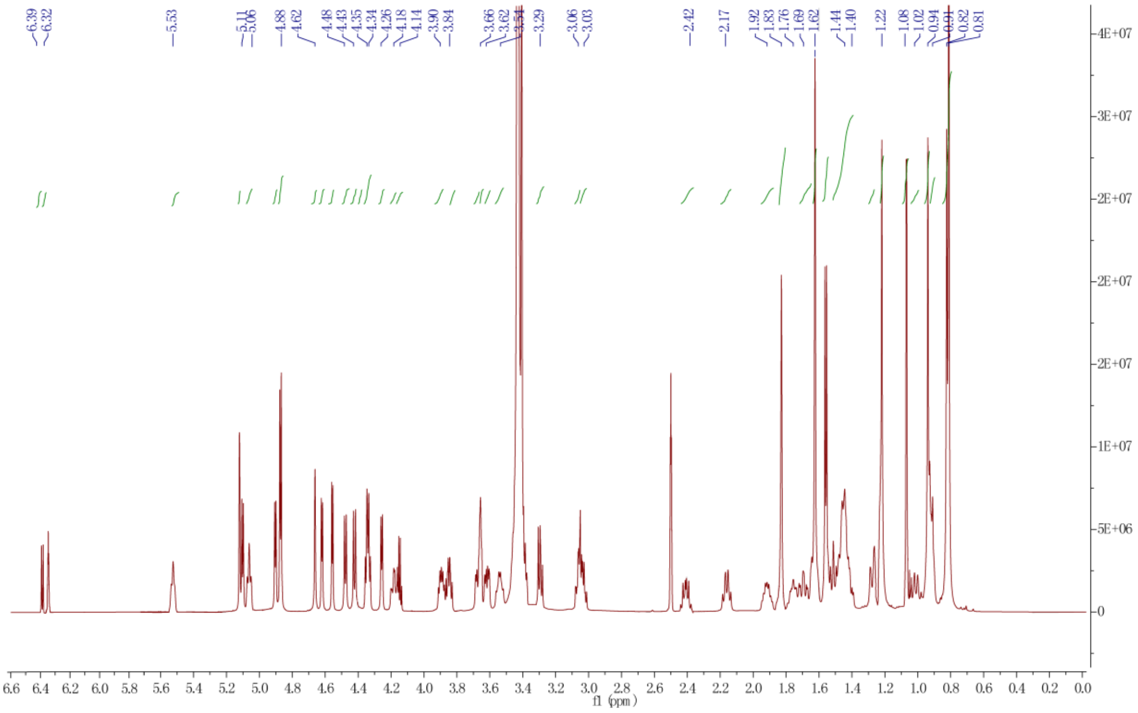

Figure S1-1. ^1H NMR spectrum of Rm1 in DMSO-D_6 (600 MHz).

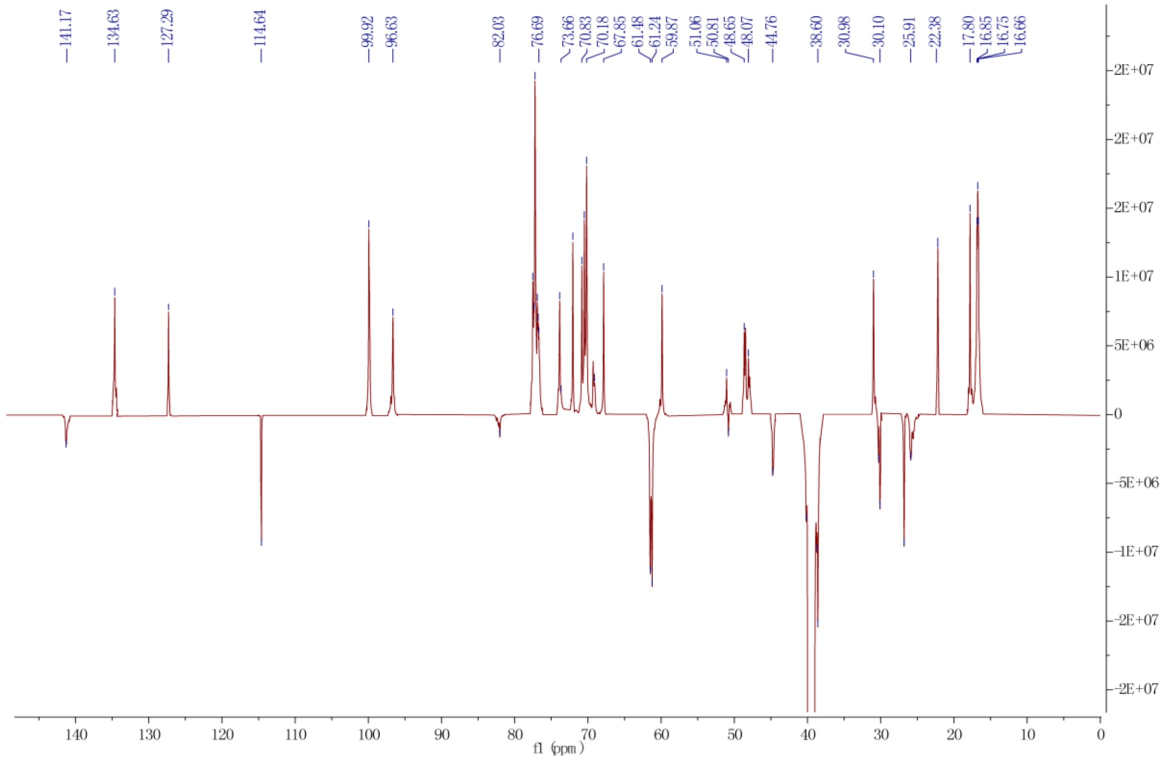

Figure S1-2. ^{13}C-DEPT NMR spectrum of Rm1 in DMSO-D$_6$ (150 MHz).

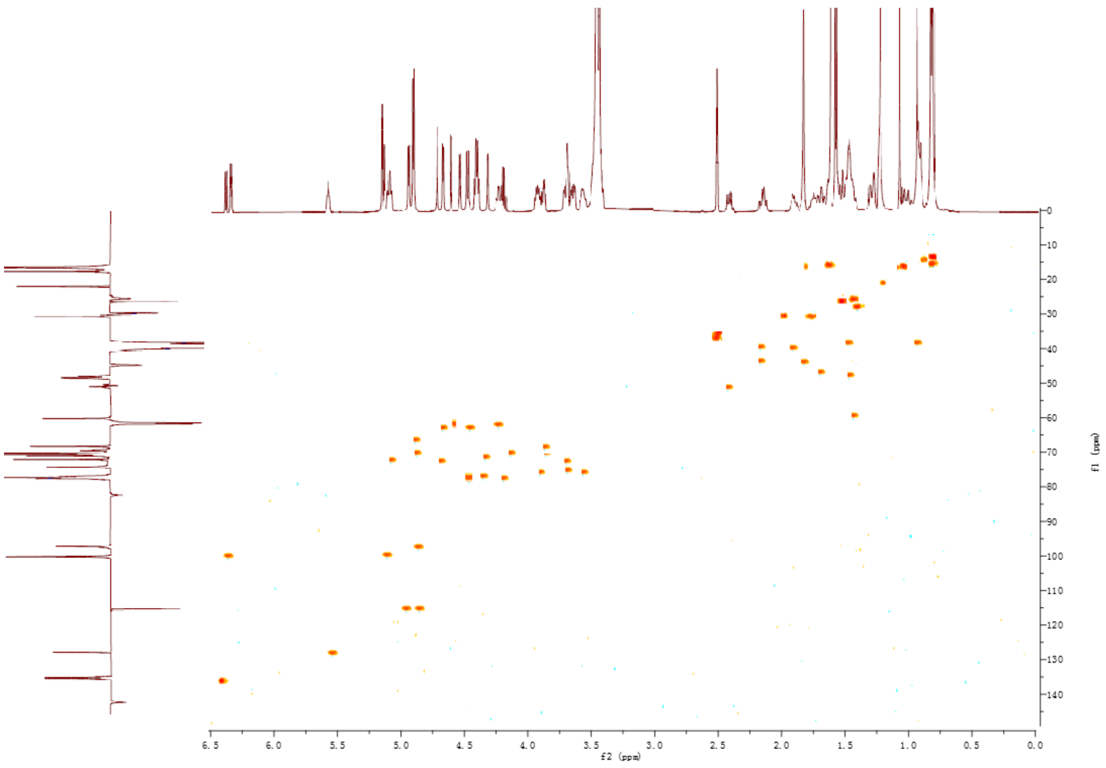

Figure S1-3. Heteronuclear multiple quantum correlation (HMQC) spectrum of Rm1.

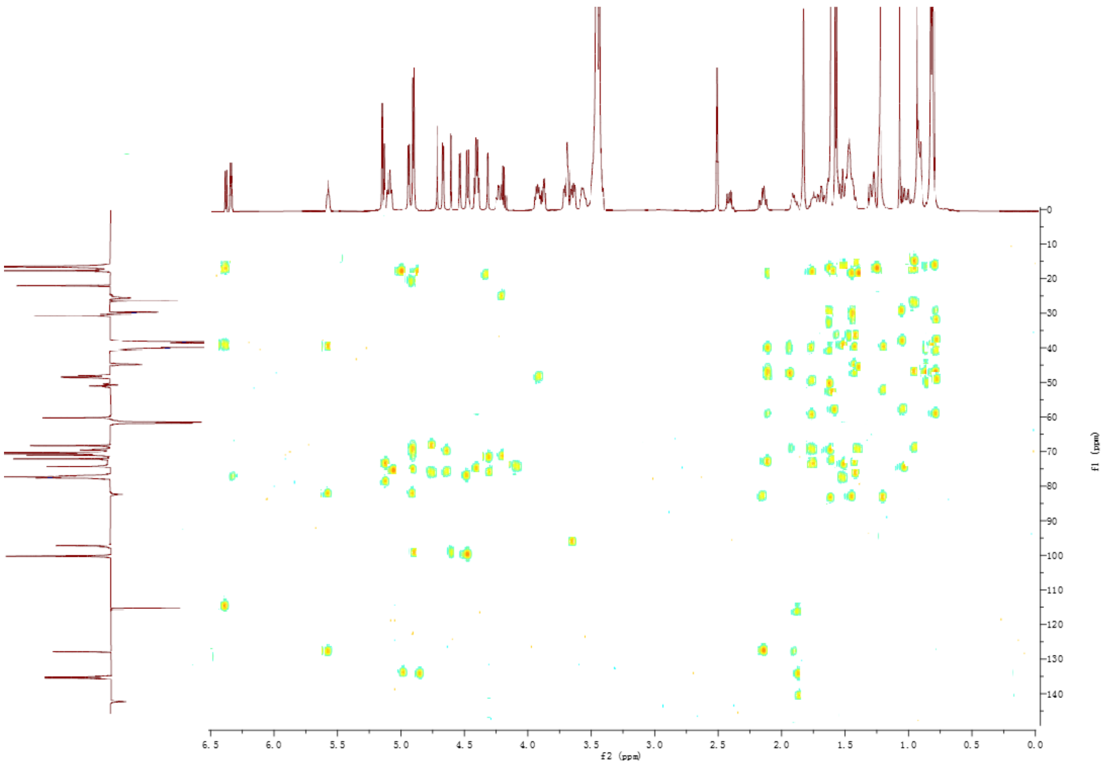

Figure S1-4. Heteronuclear multiple bond coherence (HMBC) spectrum of Rm1.

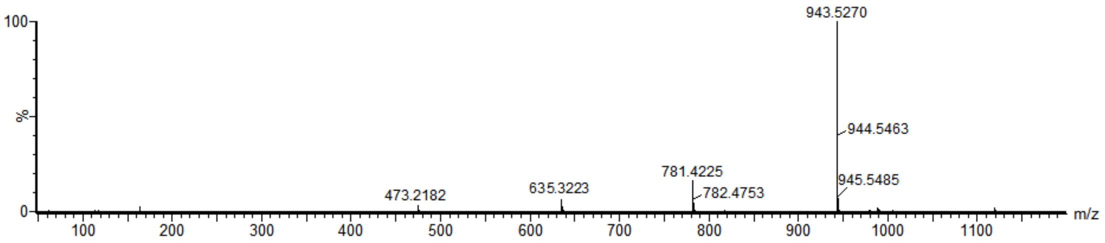

Figure S1-5. High resolution electrospray ionization mass spectroscopy (HRESIMS) spectrum of MS^E fragment ions of Rm1.

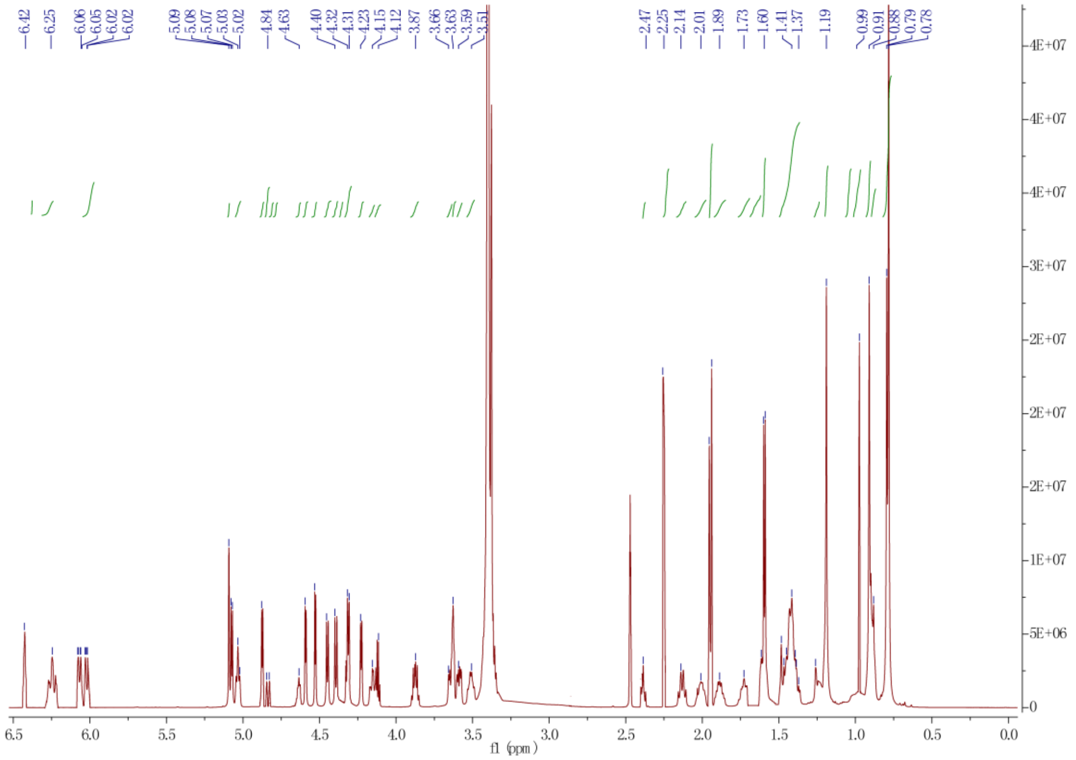

Figure S2-1. ^{1}H NMR spectrum of Rm2 in DMSO-D$_6$ (600 MHz).

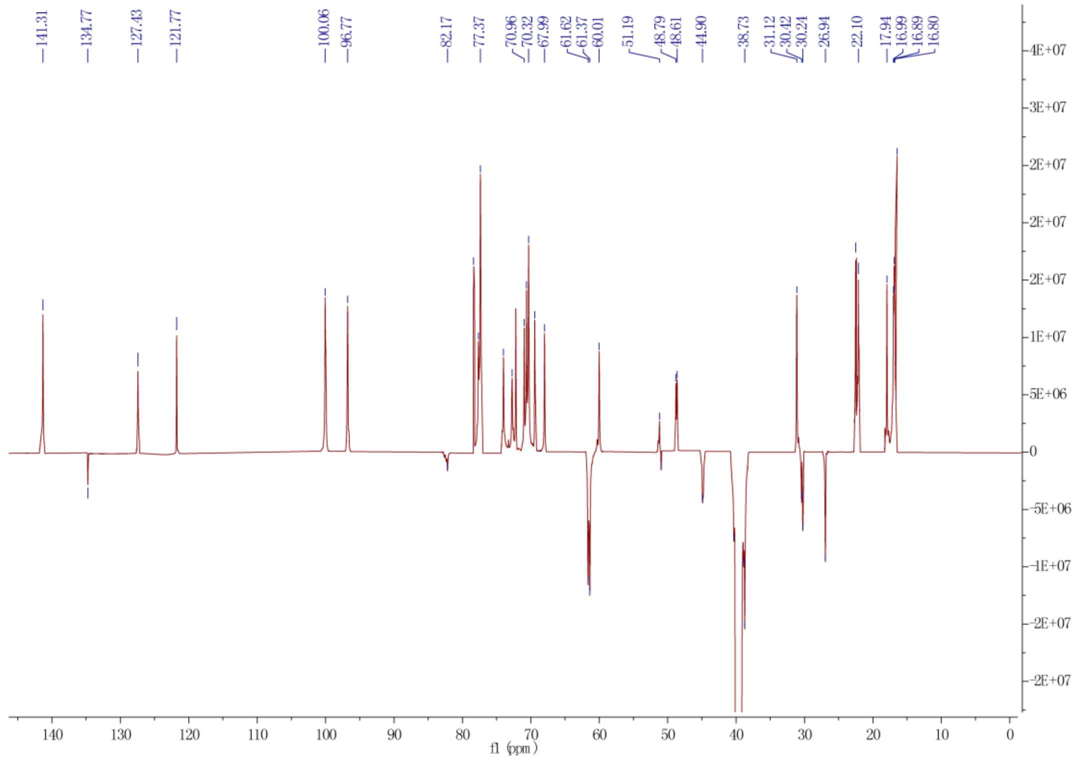

Figure S2-2. ^{13}C-DEPT NMR spectrum of Rm2 in DMSO-D$_6$ (150 MHz).

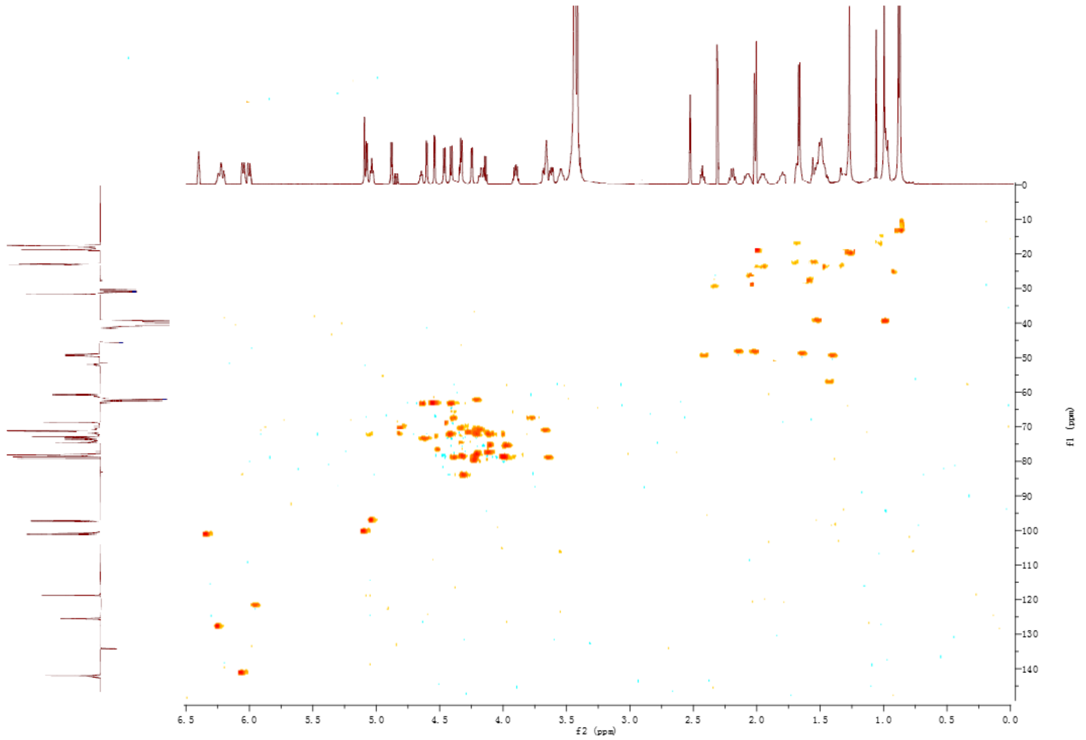

Figure S2-3. Heteronuclear multiple quantum correlation (HMQC) spectrum of Rm2.

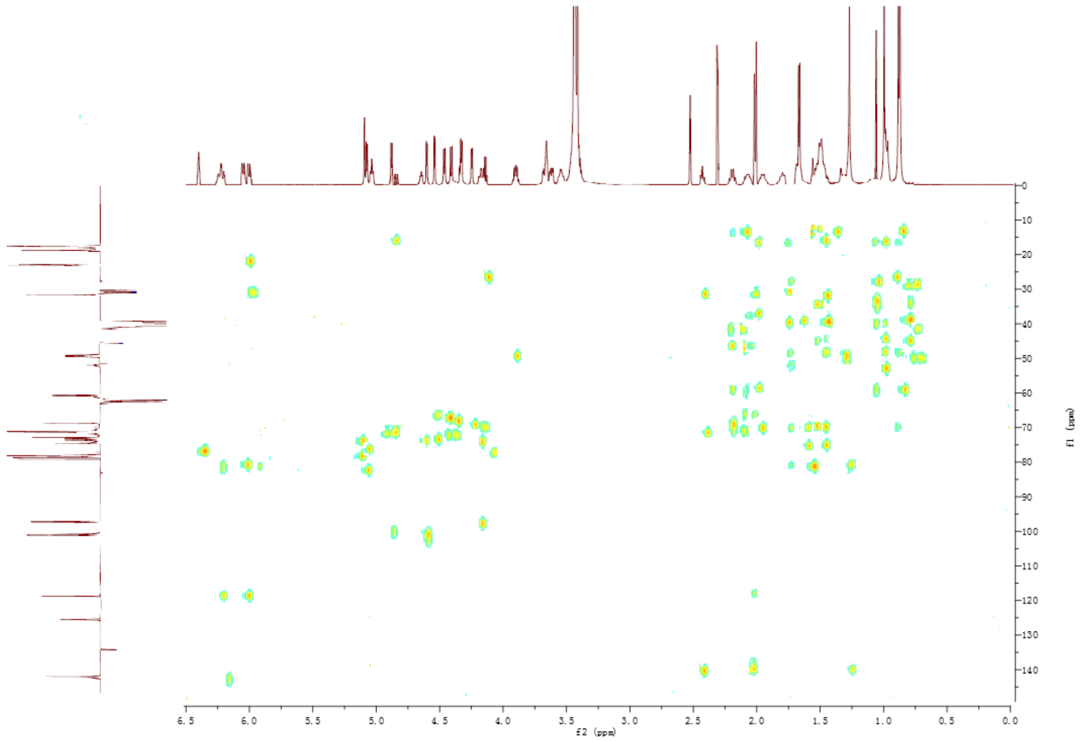

Figure S2-4. Heteronuclear multiple bond coherence (HMBC) spectrum of Rm2.

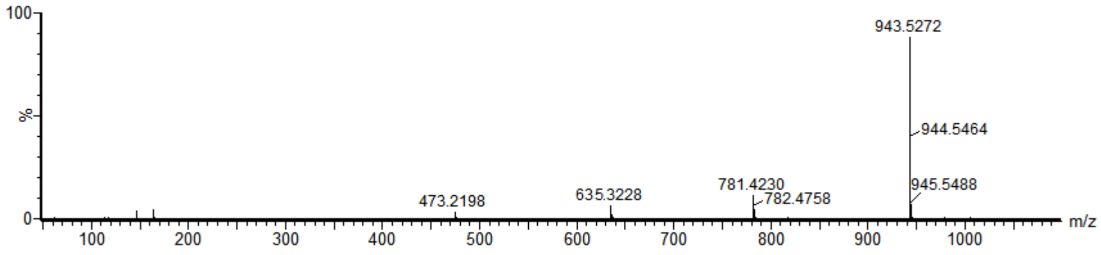

Figure S2-5. High resolution electrospray ionization mass spectroscopy (HRESIMS) spectrum of MS_E fragment ions of Rm2.

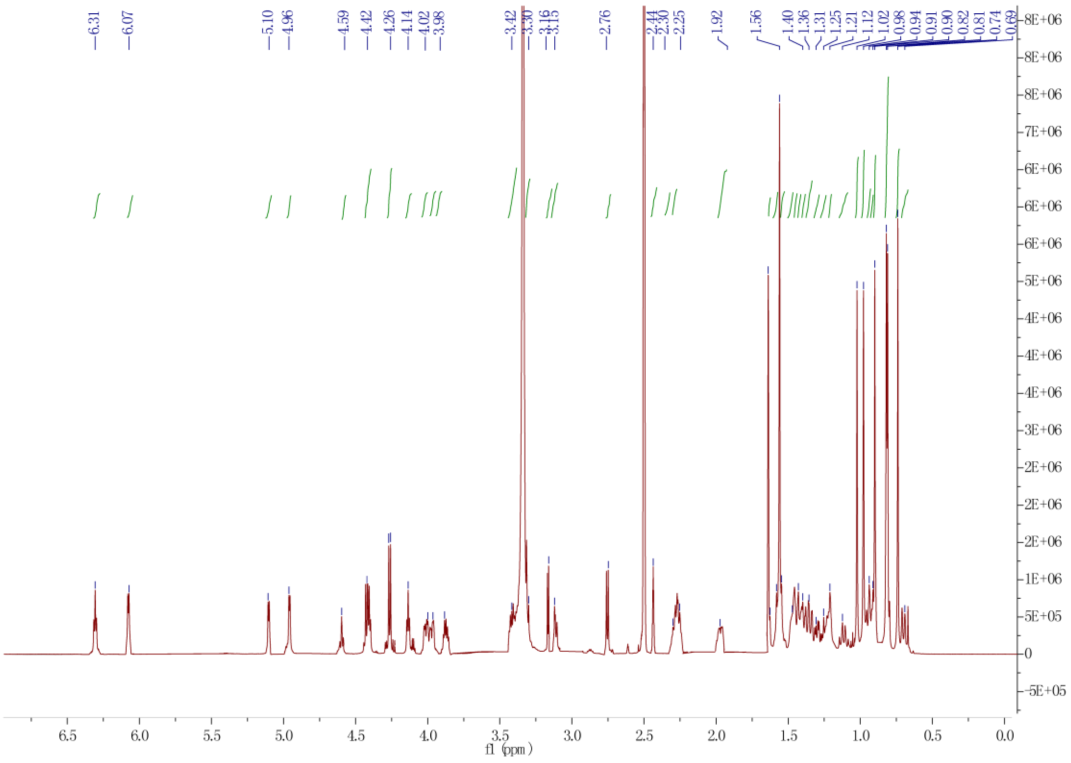

Figure S3-1. ^1H NMR spectrum of Rm3 in DMSO-D_6 (600 MHz).

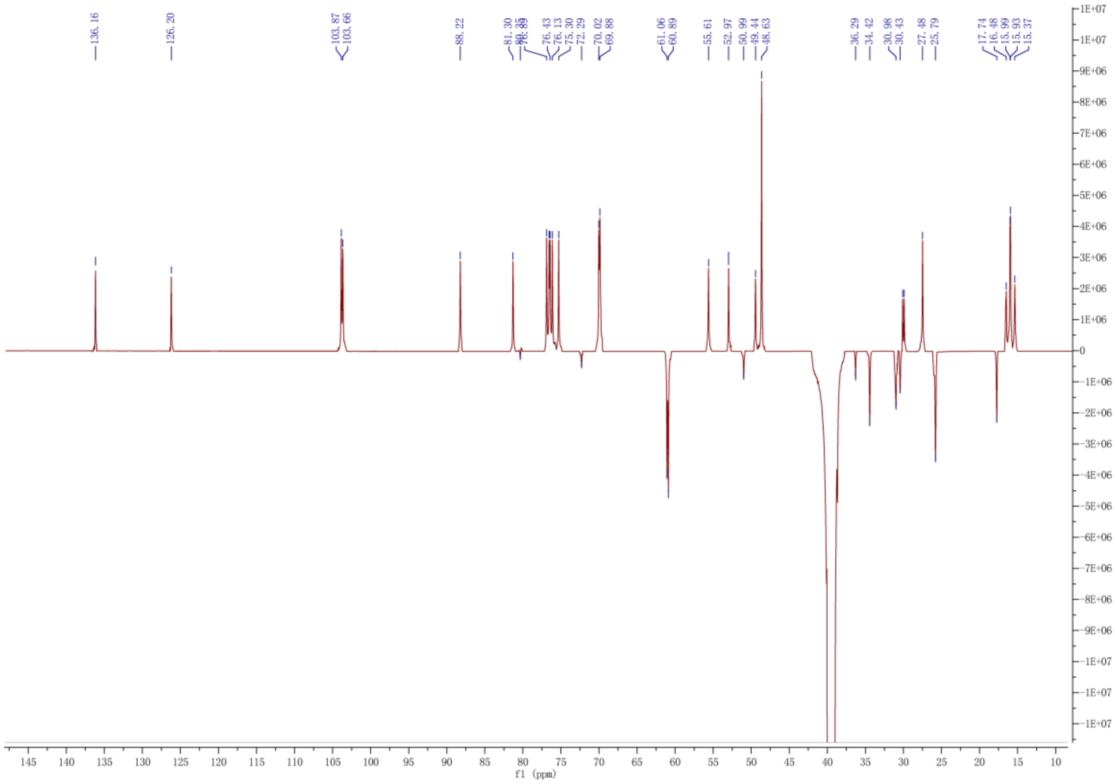

Figure S3-2. ^{13}C-DEPT NMR spectrum of Rm3 in DMSO-D_6 (150 MHz).

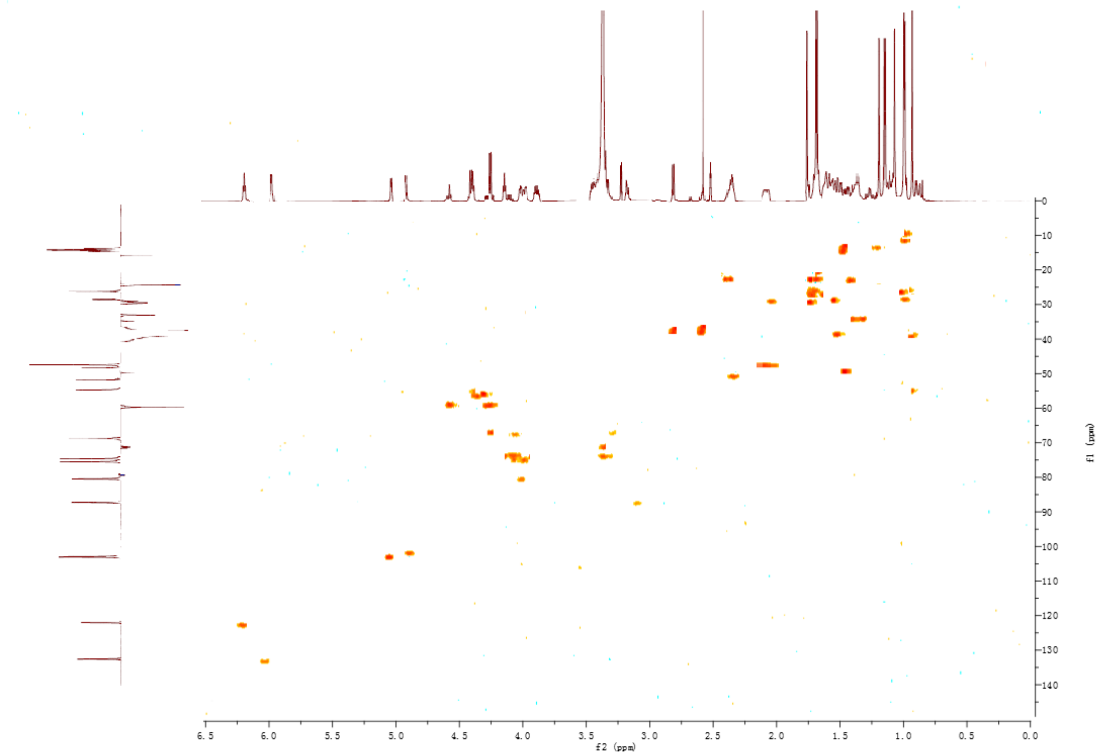

Figure S3-3. Heteronuclear multiple quantum correlation (HMQC) spectrum of Rm3.

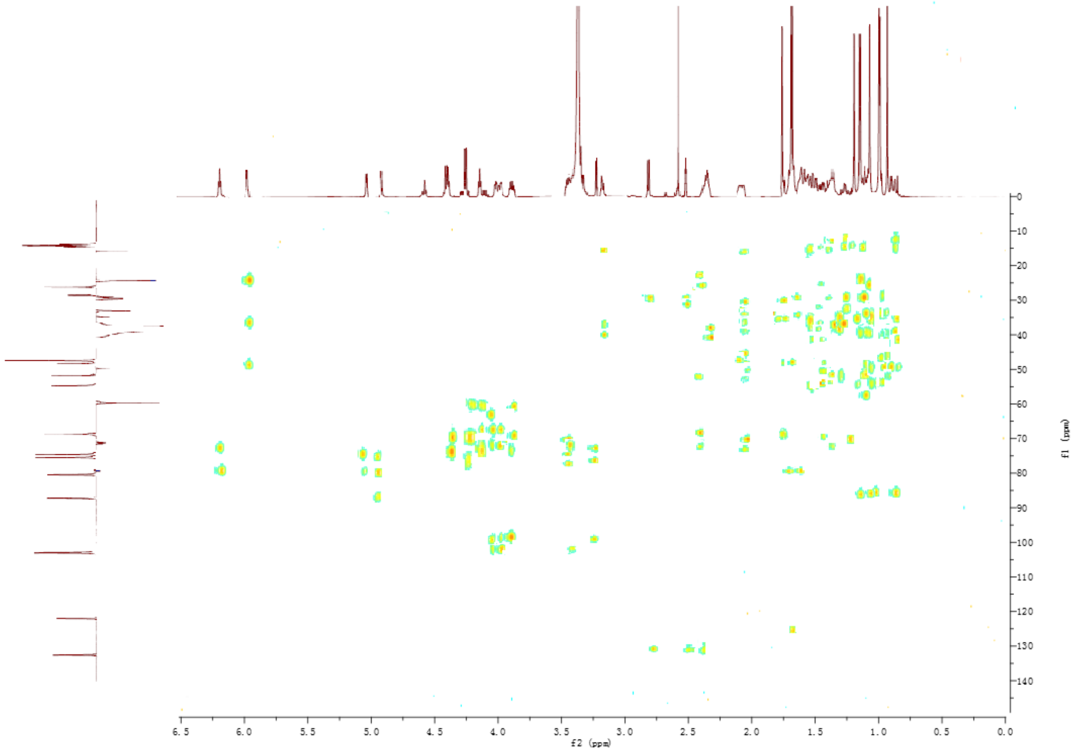

Figure S3-4. Heteronuclear multiple bond coherence (HMBC) spectrum of Rm3.

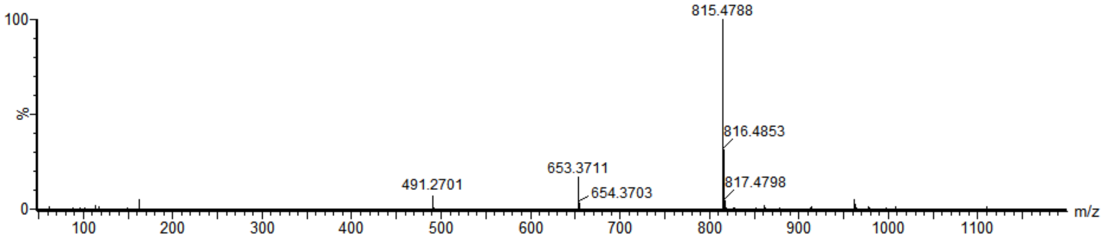

Figure S3-5. High resolution electrospray ionization mass spectroscopy (HRESIMS) spectrum of MS_E fragment ions of Rm3.

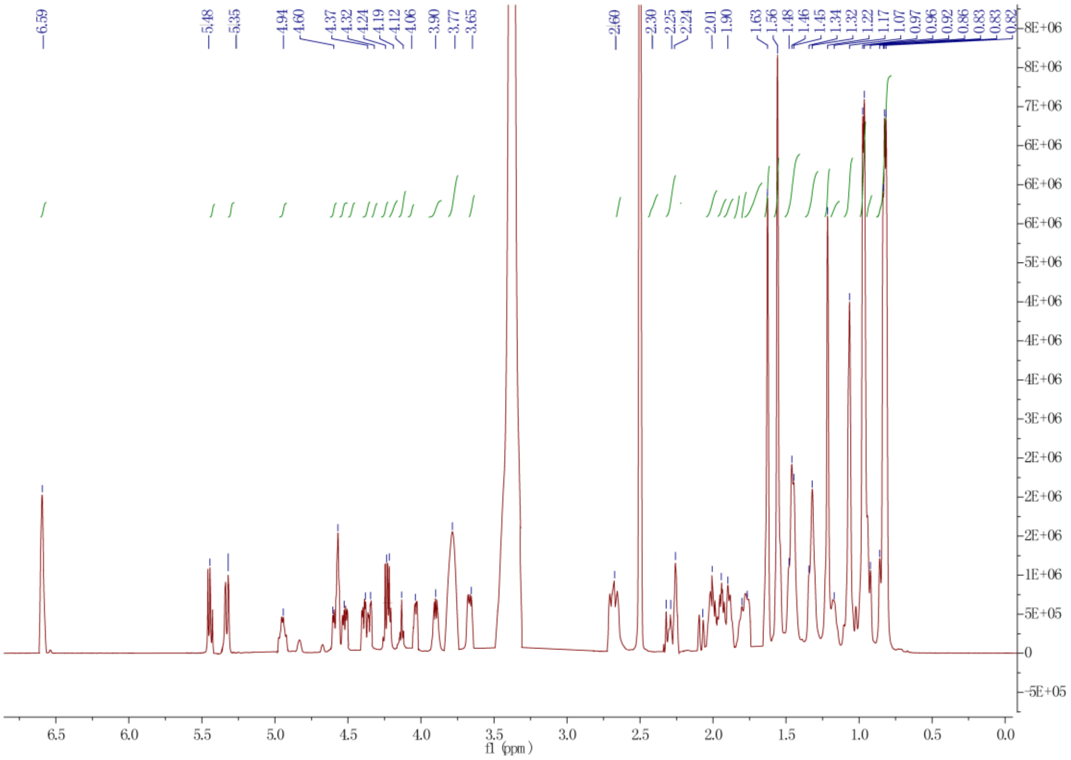

Figure S4-1. ^{1}H NMR spectrum of Rm4 in DMSO-D_6 (600 MHz).

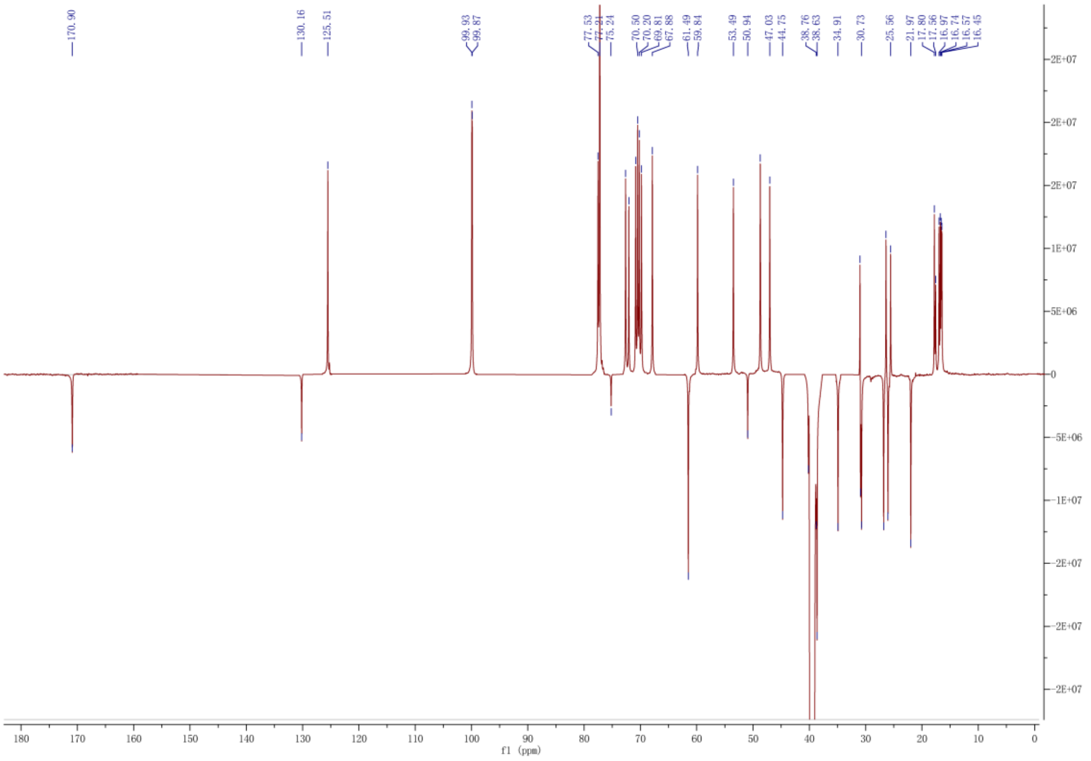

Figure S4-2. ^{13}C-DEPT NMR spectrum of Rm4 in DMSO-D_6 (150 MHz).

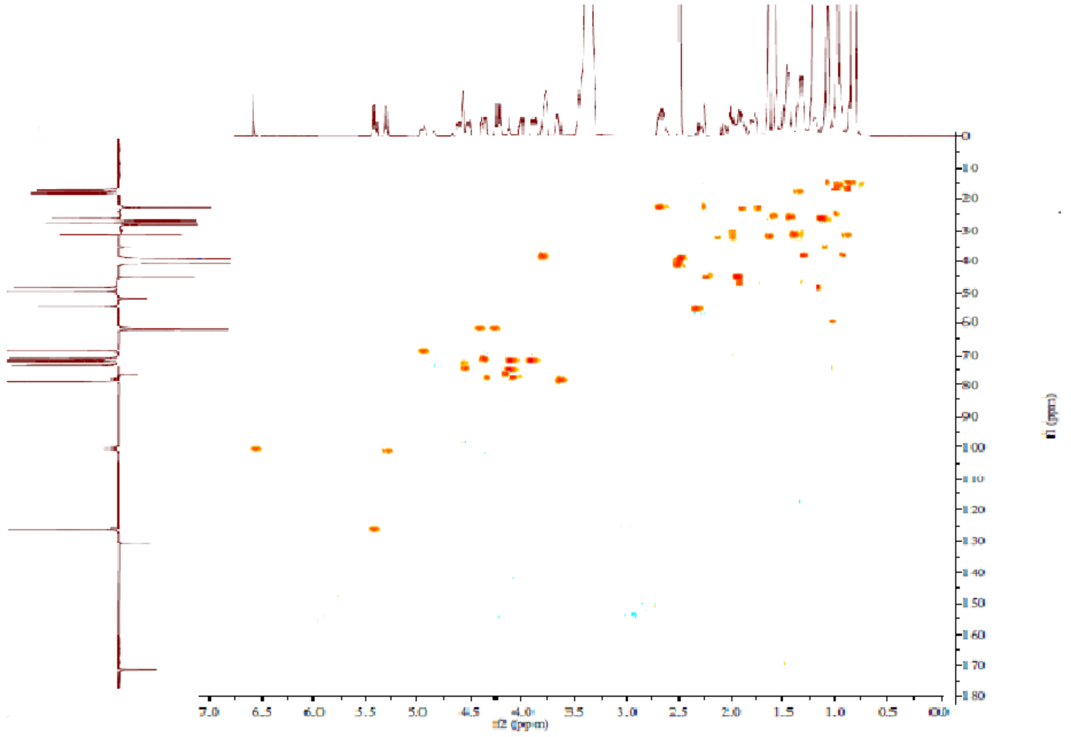

Figure S4-3. Heteronuclear multiple quantum correlation (HMQC) spectrum of Rm4.

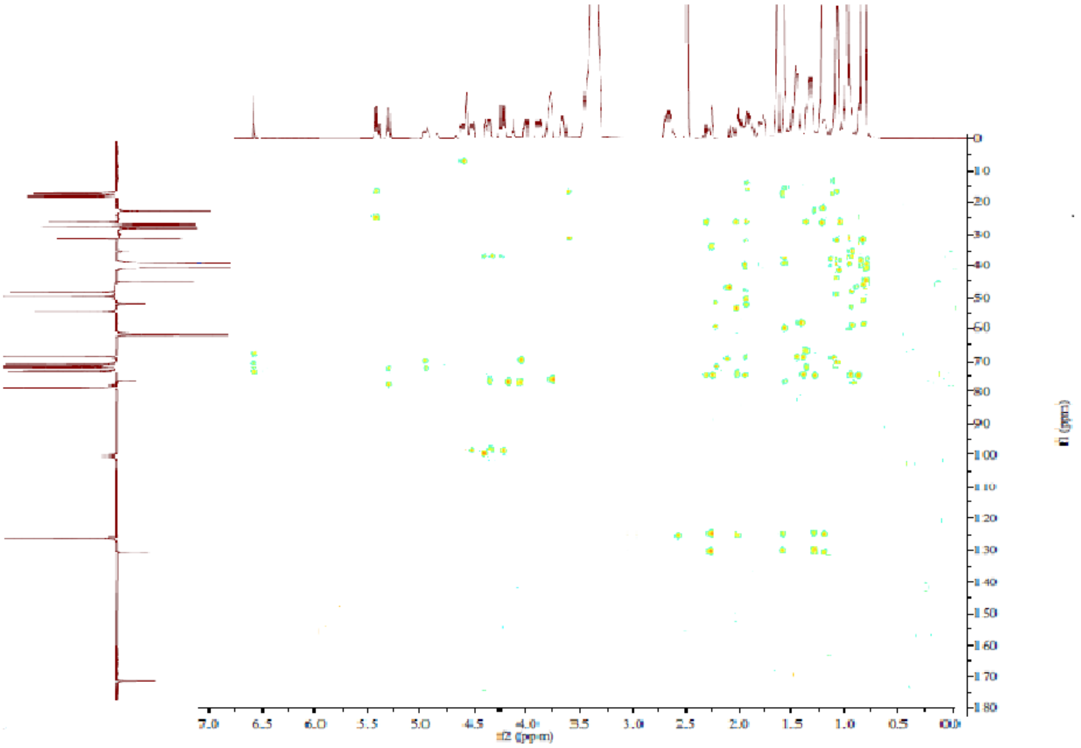

Figure S4-4. Heteronuclear multiple bond coherence (HMBC) spectrum of Rm4.

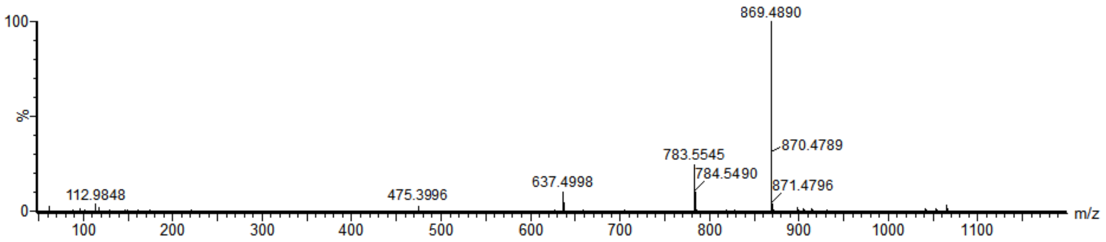

Figure S4-5. High resolution electrospray ionization mass spectroscopy (HRESIMS) spectrum of MS_E fragment ions of Rm4.

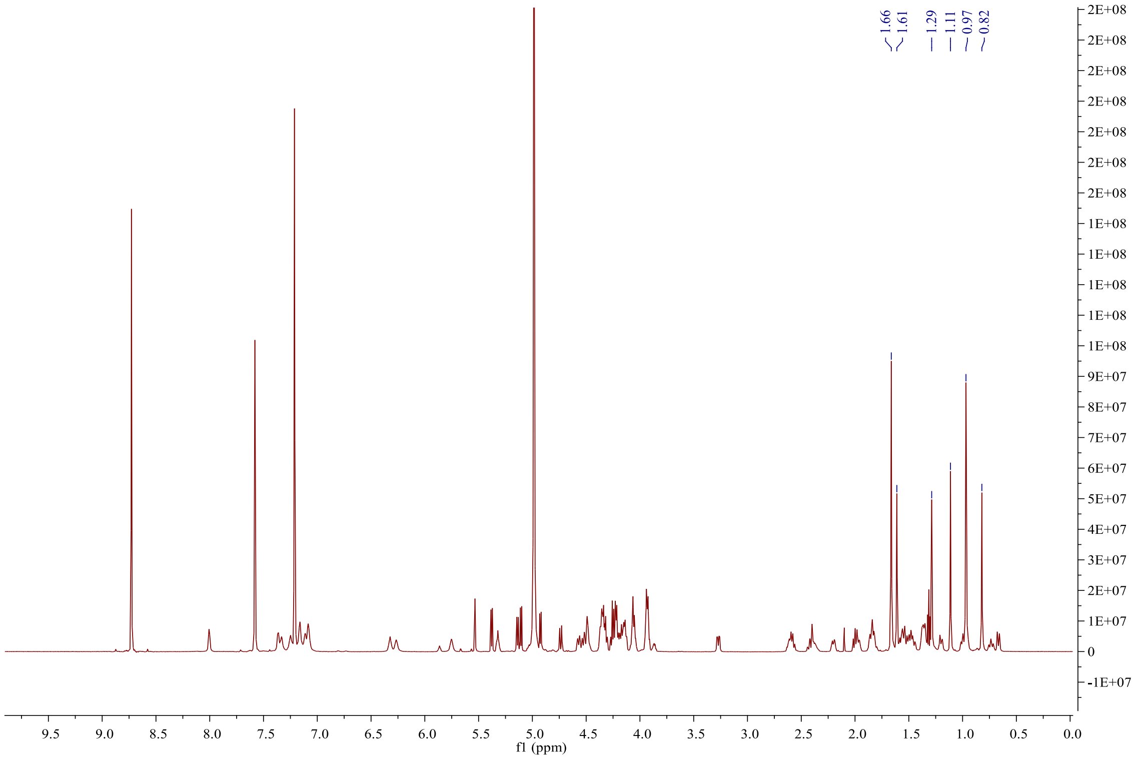

Figure S5-1. ^{1}H NMR spectrum of Rb2 in Pyridine-D_5(600 MHz).

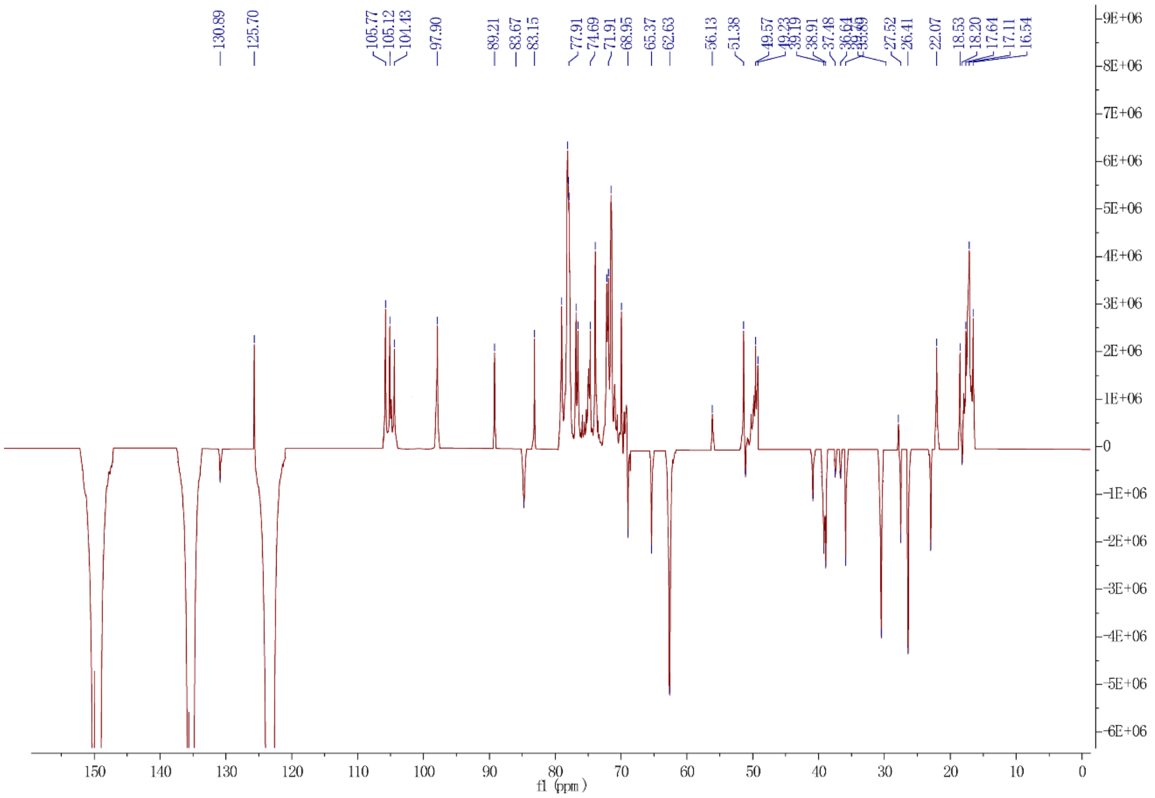

Figure S5-2. ^{13}C-DEPT NMR spectrum of Rb2 in Pyridine-D_5 (150 MHz).

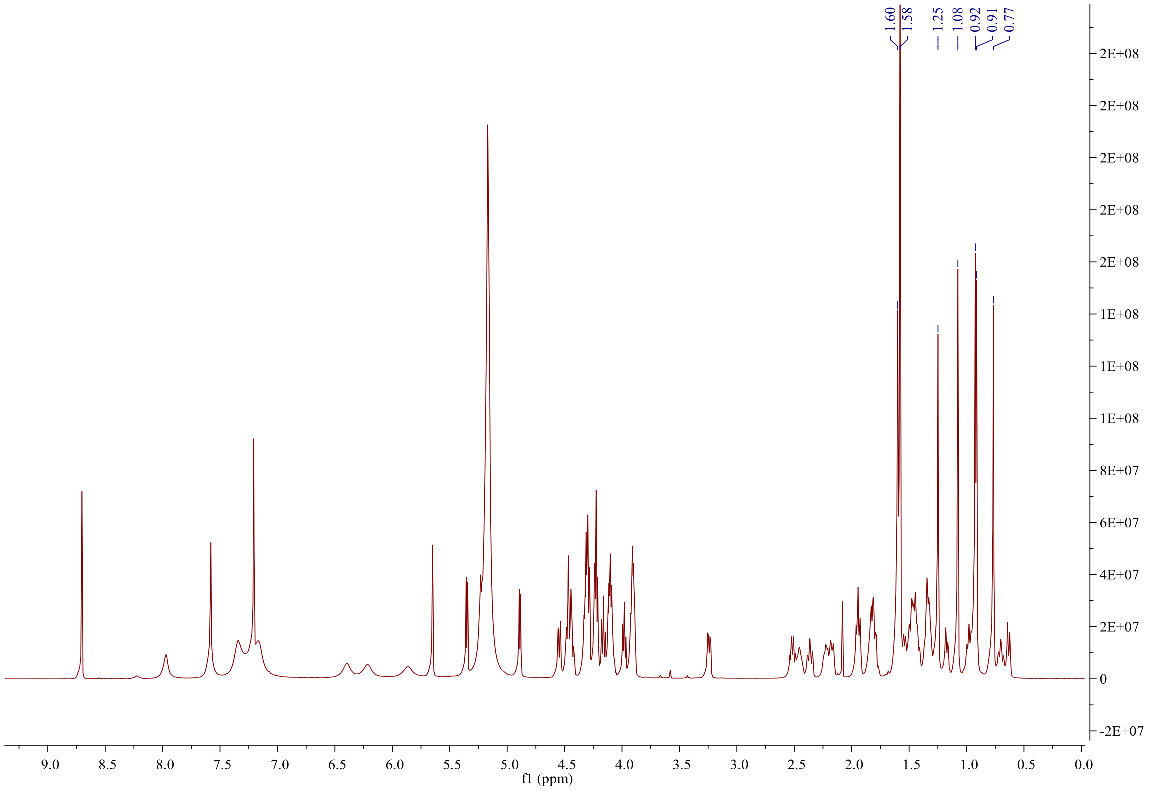

Figure S6-1. ^{1}H NMR spectrum of Rd in Pyridine-D_5(600 MHz).

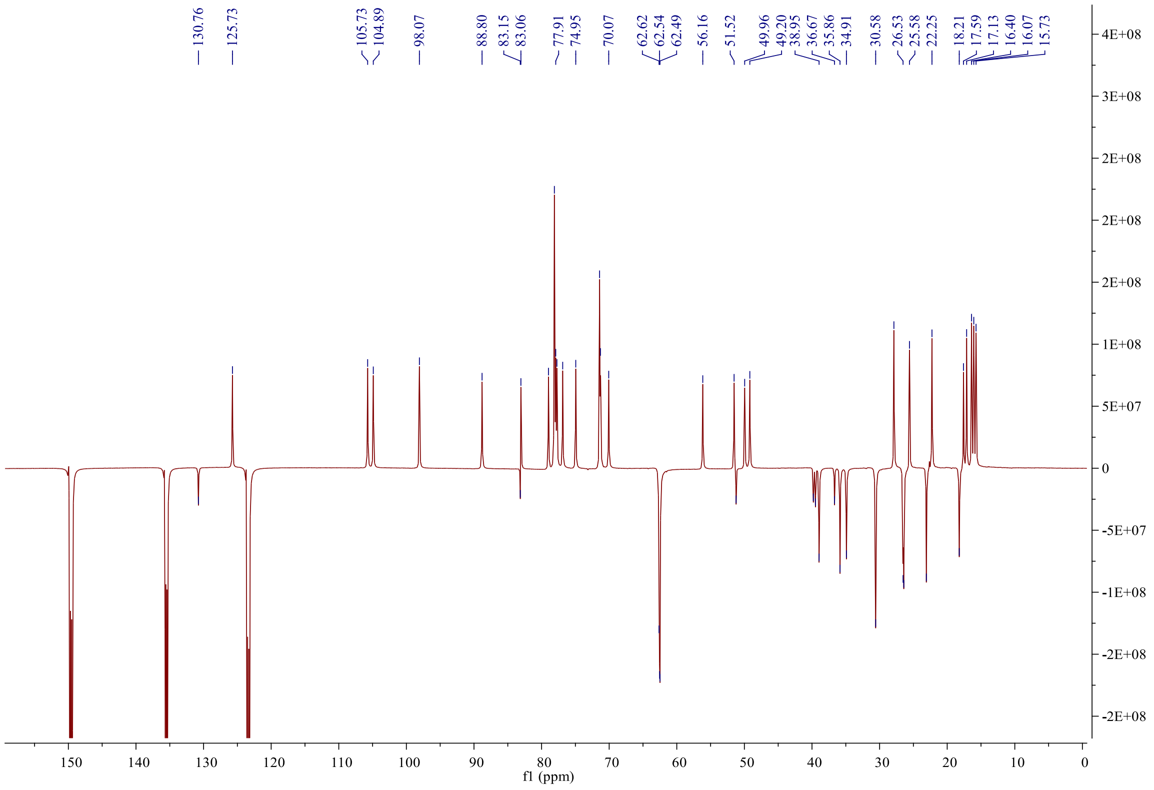

Figure S6-2. ^{13}C-DEPT NMR spectrum of Rd in Pyridine-D_5 (150 MHz).

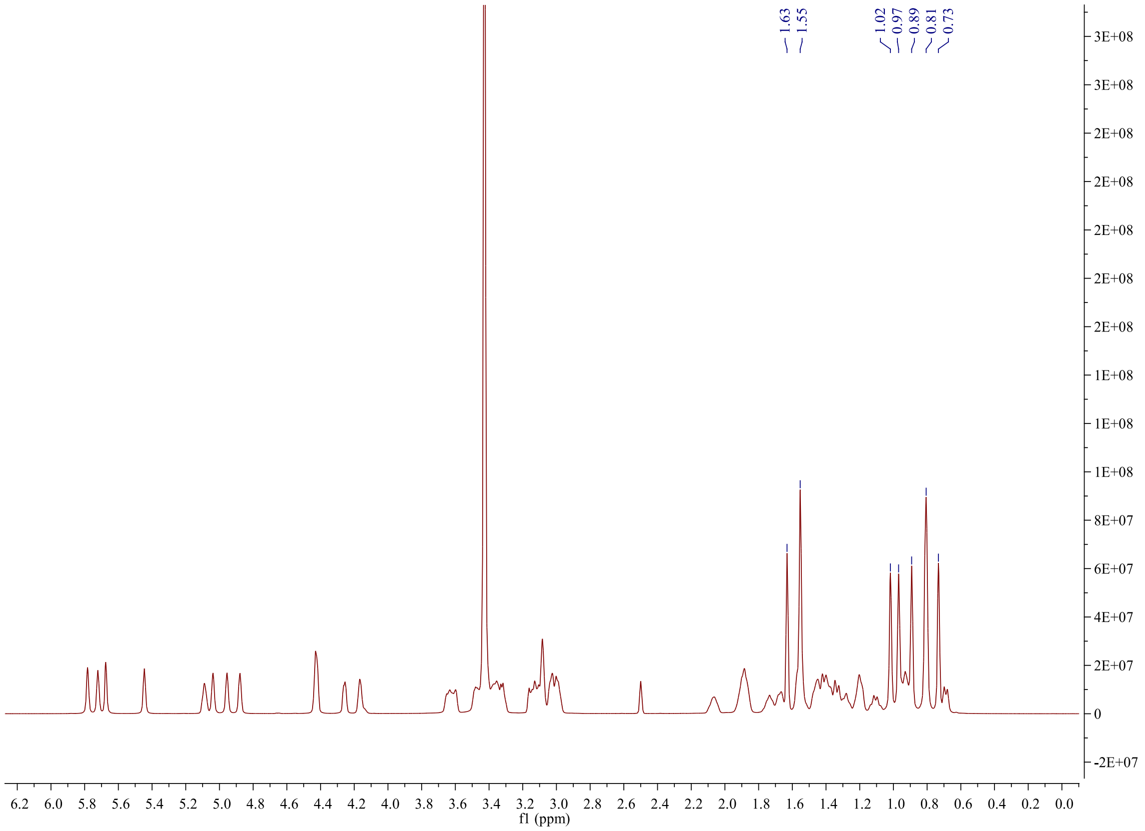

Figure S7-1. ^{1}H NMR spectrum of Rg3 in DMSO-D_6 (600 MHz).

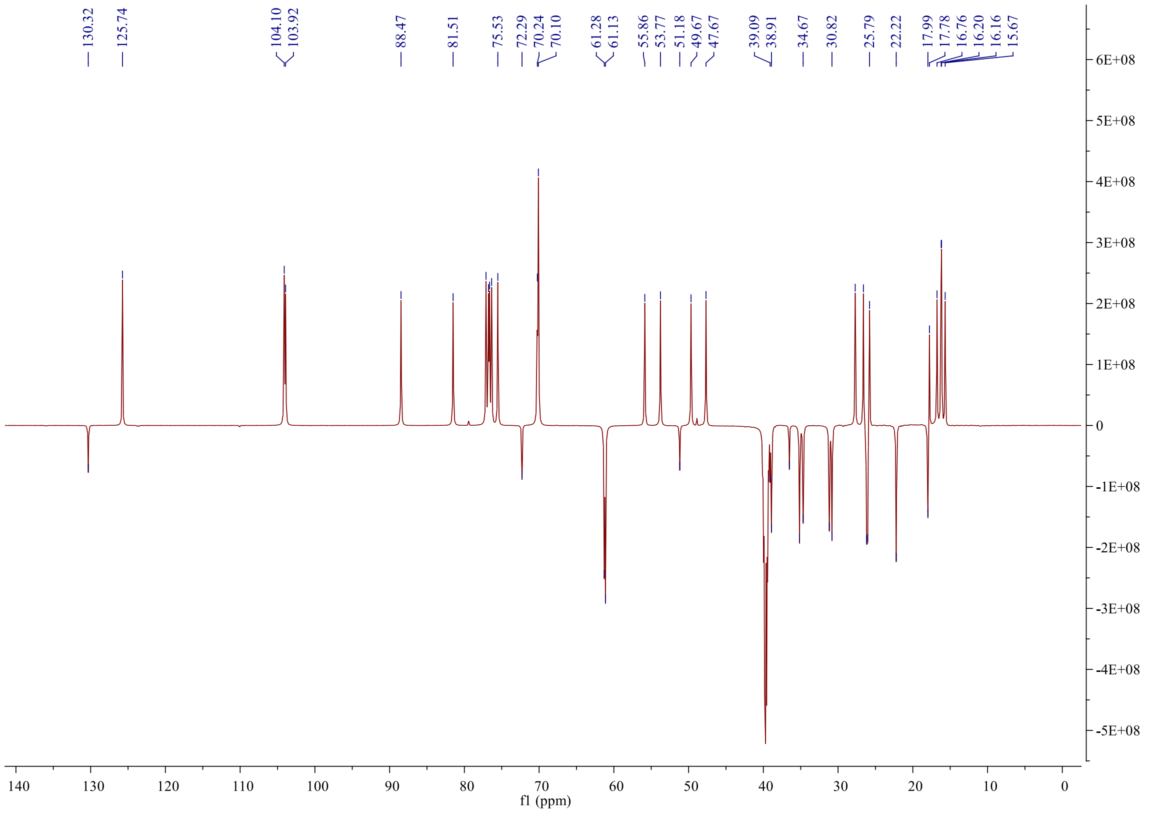

Figure S7-2. ^{13}C-DEPT NMR spectrum of Rd in DMSO-D$_6$ (150 MHz).

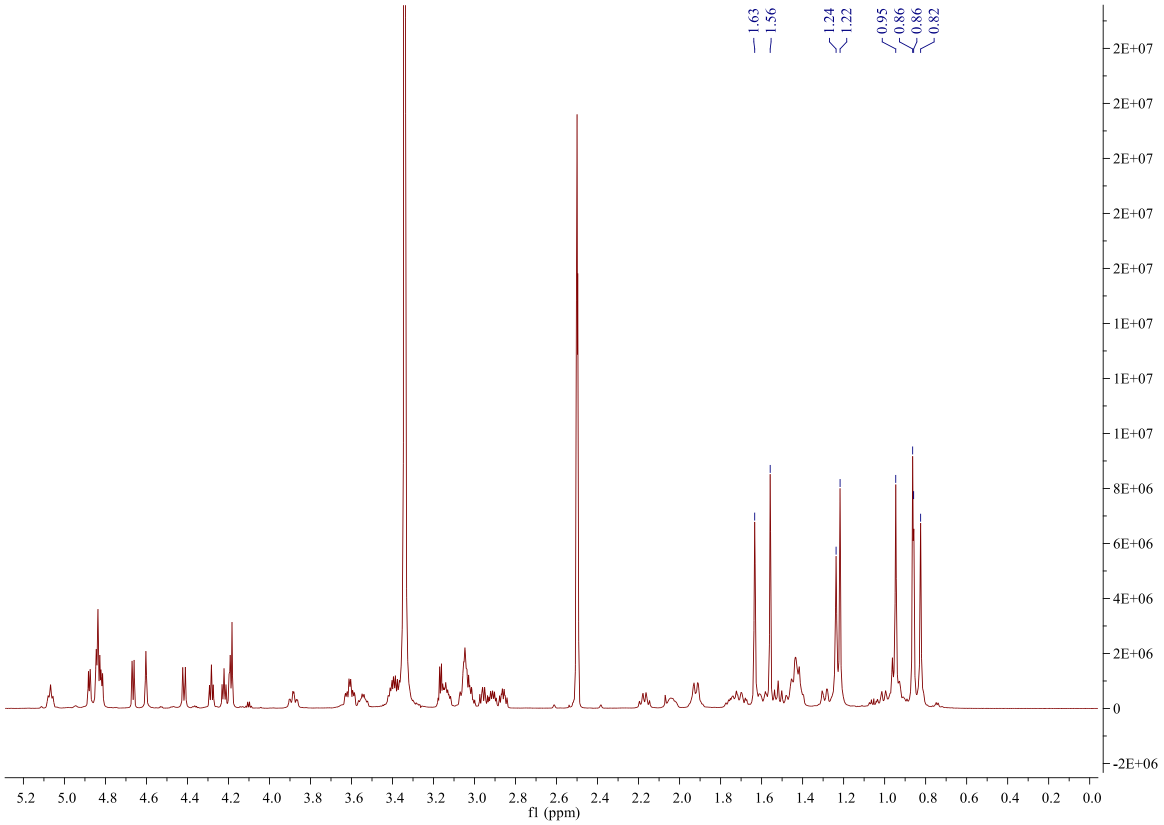

Figure S8-1. ^{1}H NMR spectrum of Rg1 in DMSO-D$_6$ (600 MHz).

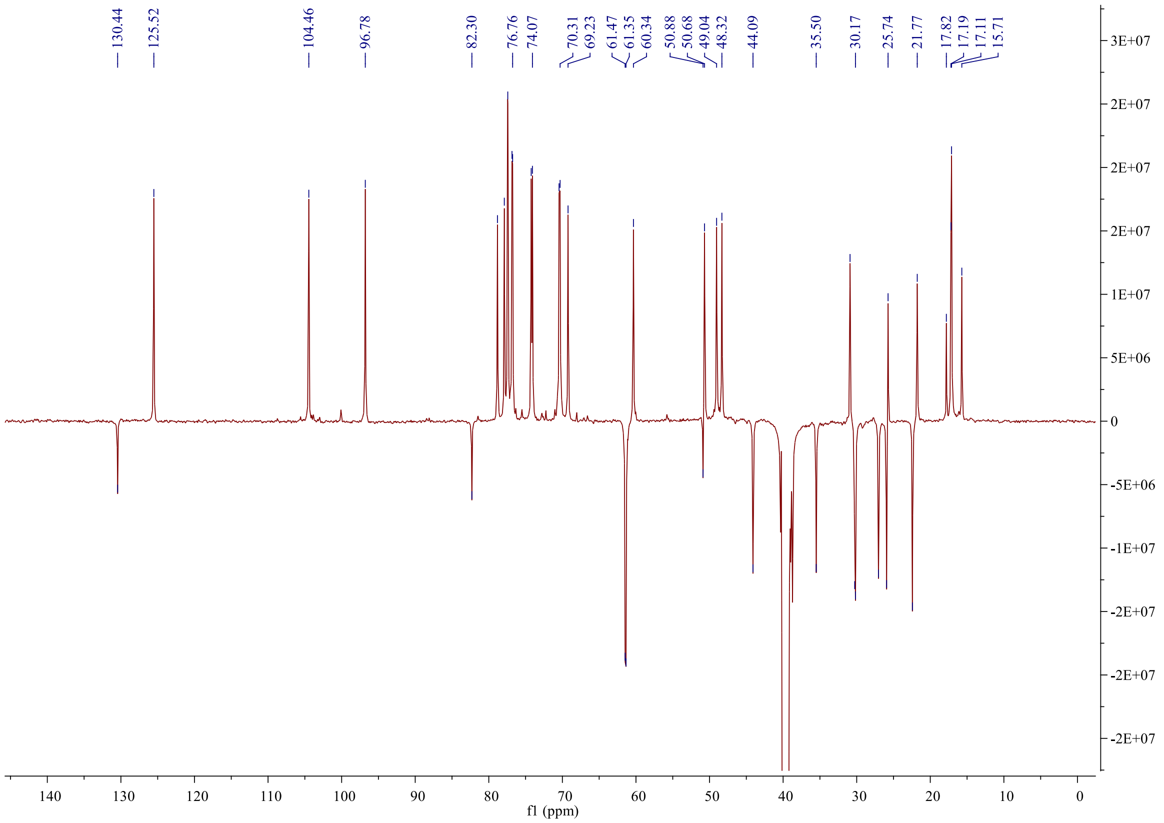

Figure S8-2. ^{13}C-DEPT NMR spectrum of Rg1 in Pyridine-D_6 (150 MHz).

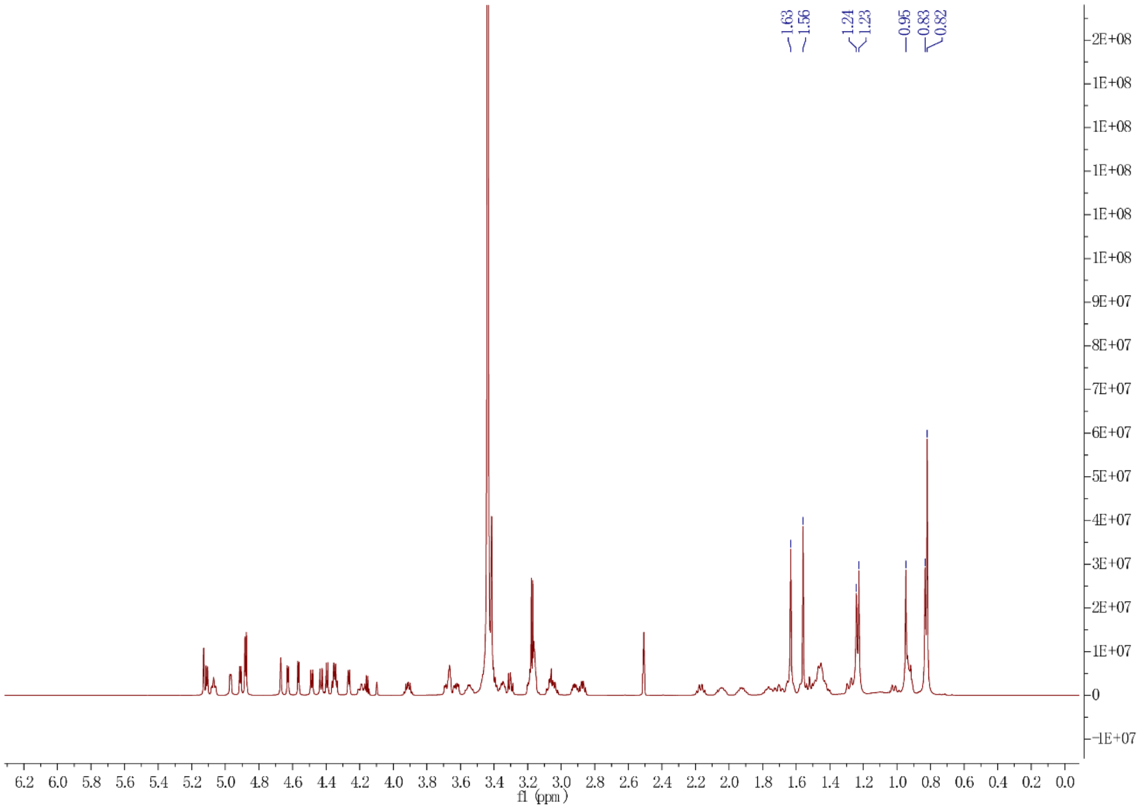

Figure S9-1. ^{1}H NMR spectrum of Rh2 in DMSO-D6 (600 MHz).

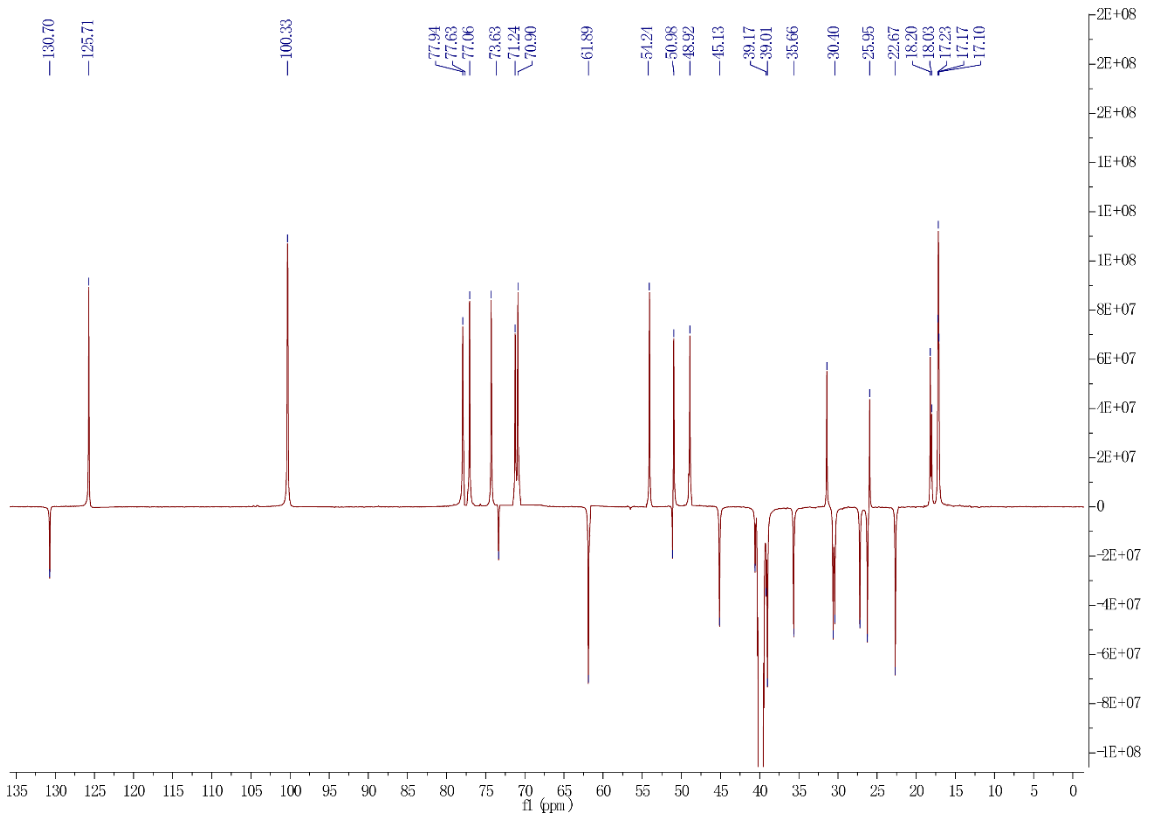

Figure S9-2. ^{13}C-DEPT NMR spectrum of Rh_2 in DMSO-D6 (150 MHz).

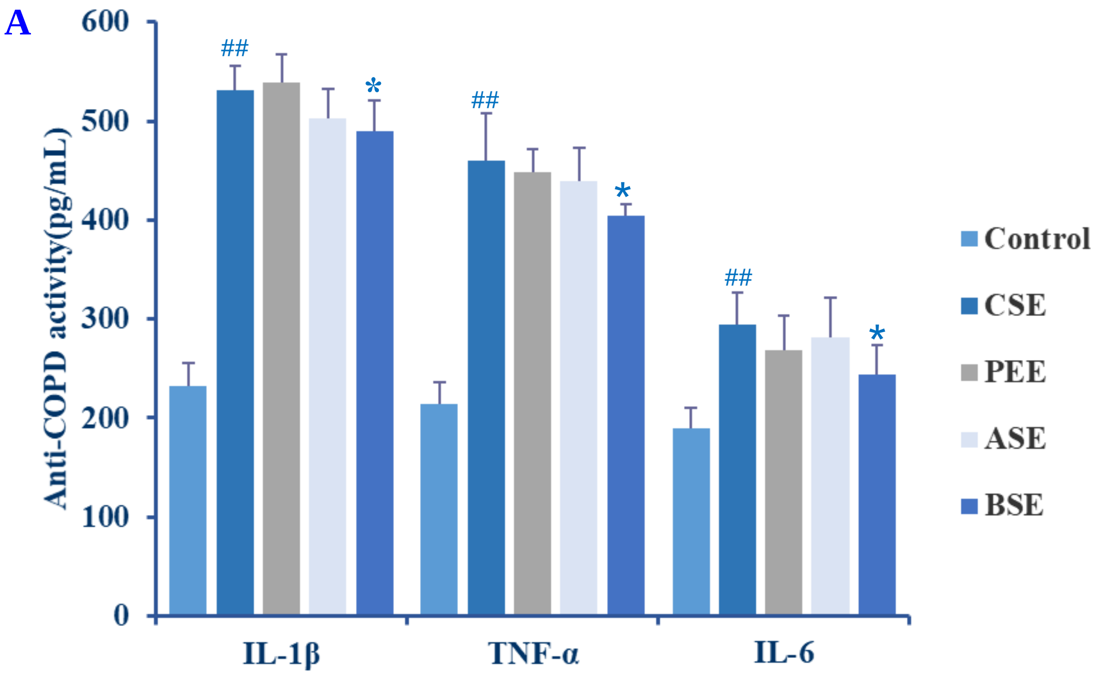

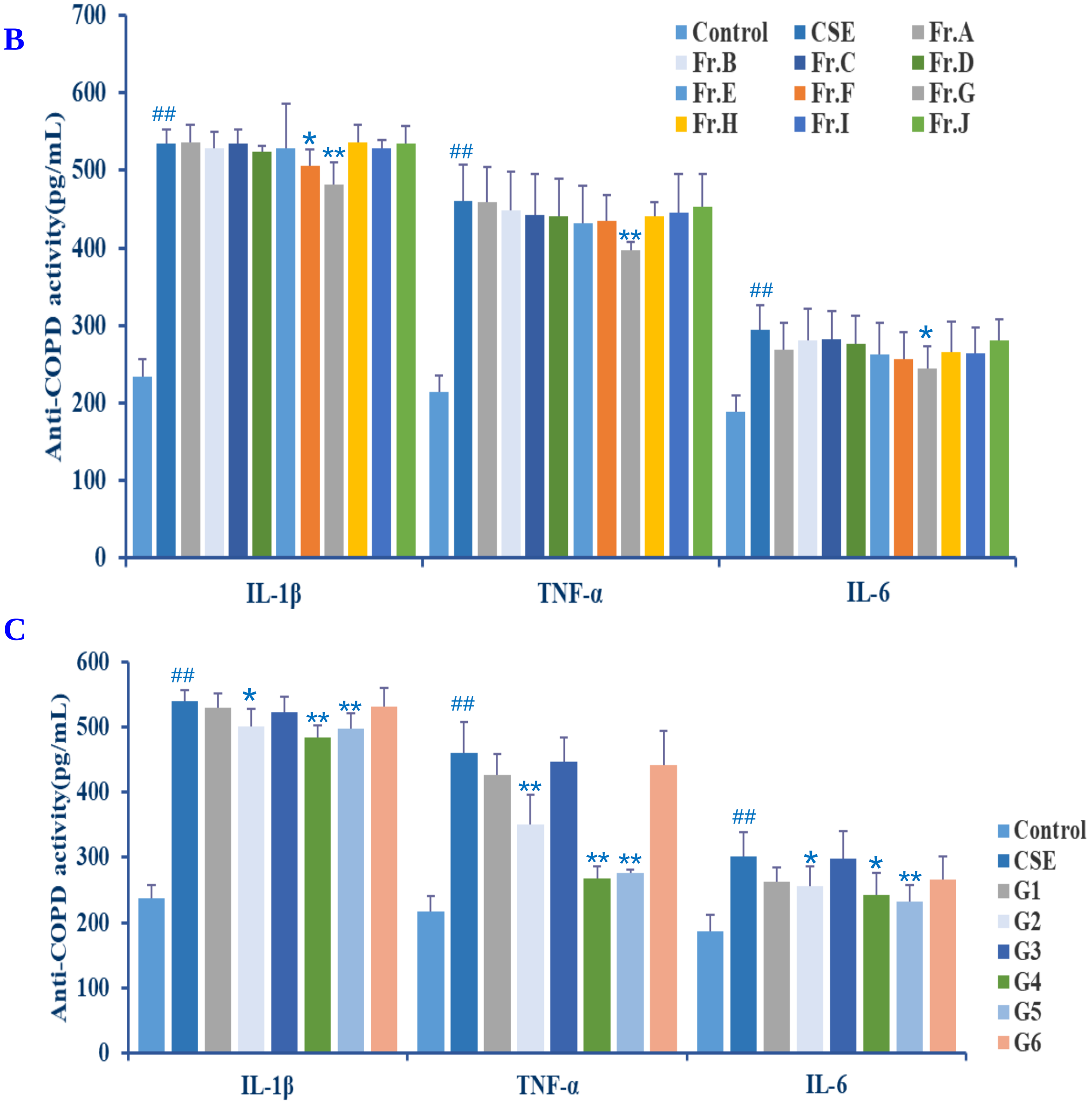

Figure S10. Anti-inflammatory effect of petroleum ether soluble extracts (PEe), ethyl acetate soluble extracts(ASe) , n-butanol soluble extracts(BSe) (200 μM)(A), FrA-J(B) and G1-6(C) on the inflammatory cytokine tumor necrosis factor-α (TNF-α), interleukin-1β (IL-1β) and interleukin-6 (IL-6) in CSE-exposed A549 cells. The results are expressed as mean ± S.D., n=6. ## $p < 0.01$, compared with control group; ** $p < 0.01$, compared with CSE group; * $p < 0.05$, compared with CSE group.
